# The CD40 agonist HERA-CD40L results in enhanced activation of antigen presenting cells, promoting an anti-tumor effect alone and in combination with radiotherapy

**DOI:** 10.3389/fimmu.2023.1160116

**Published:** 2023-05-26

**Authors:** Jamie Frankish, Debayan Mukherjee, Erminia Romano, Katharina Billian-Frey, Matthias Schröder, Karl Heinonen, Christian Merz, Mauricio Redondo Müller, Christian Gieffers, Oliver Hill, Meinolf Thiemann, Jamie Honeychurch, Tim Illidge, Jaromir Sykora

**Affiliations:** ^1^ Apogenix AG, Heidelberg, Germany; ^2^ Targeted Therapy Group, Division of Cancer Sciences, Faculty of Biology Medicine and Health, The University of Manchester, Manchester, United Kingdom

**Keywords:** CD40, HERA-CD40L, TNFRSF, TRAF2, tumor micro environment (TME), antigen presenting cells, anti-tumor responses, radiotherapy

## Abstract

**Introduction:**

The ability to modulate and enhance the anti-tumor immune responses is critical in developing novel therapies in cancer. The Tumor Necrosis Factor (TNF) Receptor Super Family (TNFRSF) are potentially excellent targets for modulation which result in specific anti-tumor immune responses. CD40 is a member of the TNFRSF and several clinical therapies are under development. CD40 signaling plays a pivotal role in regulating the immune system from B cell responses to myeloid cell driven activation of T cells. The CD40 signaling axis is well characterized and here we compare next generation HERA-Ligands to conventional monoclonal antibody based immune modulation for the treatment of cancer.

**Methods & results:**

HERA-CD40L is a novel molecule that targets CD40 mediated signal transduction and demonstrates a clear mode of action in generating an activated receptor complex via recruitment of TRAFs, cIAP1, and HOIP, leading to TRAF2 phosphorylation and ultimately resulting in the enhanced activation of key inflammatory/survival pathway and transcription factors such asNFkB, AKT, p38, ERK1/2, JNK, and STAT1 in dendritic cells. Furthermore, HERA-CD40L demonstrated a strong modulation of the tumor microenvironment (TME) via the increase in intratumoral CD8+ T cells and the functional switch from pro-tumor macrophages (TAMs) to anti-tumor macrophages that together results in a significant reduction of tumor growth in a CT26 mouse model. Furthermore, radiotherapy which may have an immunosuppressive modulation of the TME, was shown to have an immunostimulatory effect in combination with HERA-CD40L. Radiotherapy in combination with HERA-CD40L treatment resulted in an increase in detected intratumoral CD4+/8+ T cells compared to RT alone and, additionally, the repolarization of TAMs was also observed, resulting in an inhibition of tumor growth in a TRAMP-C1 mouse model.

**Discussion:**

Taken together, HERA-CD40L resulted in activating signal transduction mechanisms in dendritic cells, resulting in an increase in intratumoral T cells and manipulation of the TME to be pro-inflammatory, repolarizing M2 macrophages to M1, enhancing tumor control.

## 1 Introduction

The ability to reinvigorate anti-tumor immune response has become increasingly important in cancer following the breakthrough of immune check-point inhibitors, which has led to the immune-oncology “revolution” (1). The development of immunomodulatory agents in cancer has resulted in a large increase in immune-targeted therapies (2). Monoclonal antibodies are a major source of these modulation approaches, and there are currently more than 3000 active clinical trials evaluating T cell modulation in the immuno-oncology space, which represents ~2/3 of all oncology trials (3, 4). Despite the therapeutic breakthrough of the immune checkpoint inhibitors (ICI), the overall response rate (ORR) across all cancer types is less than 20%. This suggests that only the minority of patients are deriving clinical benefit from ICI and that novel immunotherapeutic approaches are required to further improve cancer outcomes.

An important family of genes in this regard is the TNF super family (TNFSF) and its cognate receptors (TNFRSF) (5, 6). While many emerging therapies in the immuno-oncology (IO) field are targeting the TNFRSF for the co-stimulation of naïve T cells (7), there are other members of the TNFRSF that have therapeutic value which are not expressed on T cells, such as CD40 (8, 9). CD40 is a transmembrane glycoprotein receptor and its agonistic ligand, CD154 (henceforth CD40L), play a major role in many immune related responses. The CD40-CD40L immune axis is primarily associated with B cells and is important for the sustained production of antibodies, antibody class switching, forming germinal centers, and producing memory B cells (10, 11). Although CD40 signaling plays a major role in B cell immunology, it is also an important co-stimulatory molecule expressed on myeloid cells, specifically all antigen presenting cells (APCs) (12). In addition to hematopoietic cells, CD40 is expressed on non-hematopoietic cells, including epithelial cells, endothelial cells and even cancer cells (13–17). While CD40 is widely expressed, CD40L expression is primarily associated with activated CD4^+^ T cells (18), and the interaction of these cells with CD40 expressing cells is vital in shaping immune responses. It has been previously demonstrated that ligation of CD40L to CD40 expressed on dendritic cells (DCs) and other APCs results in potent inflammatory responses and is important for the induction of CD8^+^ T cells and the activation of the adaptive immune response (19, 20), which is especially important in anti-tumor immunological responses. Given that CD40 provides a bridge between innate immune cells and the initiation of adaptive immunity, it represents an attractive target for drug development and several CD40 focused targeted therapies have undergone clinical development (8, 21). Among these candidates is Selicrelumab (also referred to as CP-870,893 or RO7009789), which is a fully human IgG2 monoclonal antibody (mAb) and is reported to have high potency. It has been postulated that this potency, alongside other mAb based therapies, is reliant on FcγR crosslinking. FcγR crosslinking causes complement dependent cytotoxicity (CDC) and antibody-dependent cell-mediated cytotoxicity (ADCC) and, while this may be the desired effect for some mAbs, as is the case for the treatment of chronic lymphocytic lymphoma (lucatumumab) or B cell lymphomas (Rituximab), it is not thought to be the desired effect for the agonism of TNFRSF. TNFRSF primarily co-stimulate the activation of pro-inflammatory transcription factors on target effector cells, such as NFκB (21). Agonistic approaches such as this have been proven to be an effective therapeutic approach, as demonstrated by completed and on-going clinical trials directly targeting the co-stimulation of T-cells, or indirectly, leading to their activation (22).

The hexavalent TNF receptor agonist (HERA) ligands (21, 23–27) comprises of two trivalent but single-chain TNFSF-receptor binding domains linked to a silenced Fc-domain. This engineering approach produces an antibody-like molecule that is not reliant on FcγR crosslinking, and has the capacity to induce true agonism of TNFRSF, such as CD40, *via* defined receptor clustering of up to 6 receptors per molecule in order to induce signal transduction, in contrast to the bivalent capacity of mAbs. Another source of major therapy in cancer is radiotherapy, which is delivered in approximately half of all cancer patients and known to have an immunoregulatory effect. Due to this well documented immune-reaction we investigated whether radiotherapy in combination with HERA-CD40L was able to further enhance or synergize the anti-tumor responses in two syngeneic mouse models of cancer.

## 2 Materials and methods

### 2.1 Engineering, expression and purification of HERA-CD40L, mmHERA-CD40L and Selicrelumab

Engineering, expression and purification of HERA-CD40L and mmHERA-CD40L was performed as described in (23). Selicrelumab was produced employing the same set of methods. The Selicrelumab sequence was retrieved from patent literature (US8388971B2; heavy chain SEQ-ID NO: 46; light chain SEQ-ID NO: 48).

### 2.2 *In vitro* biological activity of CD40 agonists

We evaluated CD40 signaling *in vitro* following treatment with HERA-CD40L or a clinical benchmark anti-human CD40 monoclonal antibody (Selicrelumab) by measuring luciferase activity in a CD40-specific cell-based bioassay (NFκB-luc2/CD40 Jurkat cell bioassay, Promega GmbH). NFκB-luc2/CD40-expressing Jurkat cells were plated in a 96-well plate and incubated briefly at 37°C prior to addition of the indicated concentrations of HERA-CD40L, or anti-CD40 monoclonal antibody. The luciferase assay reagent was added and luminescence (RLU) was measured (Tecan Infinite F500). Cross-linking (X-Link) was achieved by adding equal amounts (titration starting at 2000 ng/ml) of rabbit-anti-human IgG Fc Dianova (Cat. No. 309-005-008).

### 2.3 Monocyte isolation, dendritic cell and macrophage culture

Monocytes were isolated from PBMCs derived from human buffy coat using negative selection (EasySep Human Monocytes Isolation Kit #19359, Stemcell). For the generation of immature dendritic cells (iDCs), isolated monocytes were cultured in RPMI 1640 medium supplemented with 10% FBS, penicillin (100 U/ml) and streptomycin (100 µg/ml) with the addition of GM-CSF for 3 days (25 ng/ml), then GM-CSF (25 ng/ml) plus IL-4 (25 ng/ml) for an additional 3 days. For the generation of macrophages, isolated monocytes were cultured in RPMI 1640 medium supplemented with 10% FBS, penicillin (100 U/ml) and streptomycin (100 µg/ml) and either treated with GM-CSF (80 ng/ml) for 7 days and on day 7 treated with IFNγ (20 ng/ml) and LPS (10 ng/ml) overnight for M1 polarized macrophages, or treated with M-CSF (20 ng/ml) for 7 days and on day 7 treated with LIF and IL-6 (20 ng/ml respectively) overnight for M2d macrophages.

### 2.4 Stimulation of iDCs and lysis for western blot analysis

Cells were stimulated at the indicated time points with HERA-CD40L and Selicrelumab (100 ng/ml or 500 ng/ml). Stimulation was halted with ice-cold PBS. Cells were washed (2x) with ice-cold PBS and were lysed in RIPA lysis and extraction buffer (ThermoFisher, Cat. No. 89901) and PMSF (Cell Signaling Cat. No. 8553) supplemented with Protease Inhibitor (cOmplete™, Mini, EDTA-free Protease Inhibitor Cocktail Sigma-Aldrich, Cat. No. 04693159001) and Phosphatase Inhibitor (PhosSTOP, Sigma-Aldrich, Cat. No. 04906837001) for 30 mins on ice with agitation. Samples were then centrifuged at 13,000 RPM. Samples were treated with NuPage Sample Buffer (Life Technologies, Cat. No. NP0007) and supplemented with 1 μl of benzonase (Milipore Cat. No. 70746-3) to degrade any presence of nucleic acid prior to denaturation at 98°C for 5 mins. Following SDS-PAGE of up to 12 μg of sample protein (NuPAGE™ 4-12%, Bis-Tris, Cat. No. NP0322PK2), samples were transferred to nitrocellulose membranes (BioRad Cat. No. 170-4270) and blocked with either 5% BSA in PBS with 0.1% Tween-20 (for phospho-proteins) or 10% milk in PBS with 0.1% Tween-20 for probing with primary and secondary antibodies (see **Supplementary Table 1**). Proteins were detected with electrochemiluminescence (ECL spray, Biozym Cat. No. K-12049-D50) using a LI-COR C-DiGit^®^ Blot Scanner (LI-COR Biosciences GmbH, Germany). If required, membranes were stripped using RevitaBlot stripping buffer (Rockland, Cat. No. MB-085-0500).

### 2.5 Subcellular protein fractionation

iDCs were harvested and stimulated with HERA-CD40L or Selicrelumab (100 ng/ml). Stimulation was halted with ice-cold PBS. Cells were washed (2x) with ice-cold PBS. Subcellular fractions were isolated using Mem-PER™ Plus Membrane Protein Extraction Kit (ThermoFisher Scientific, Cat. No. 8942). Cytosolic proteins were isolated using the aforementioned kit, then adding PMSF (Cell Signaling Cat. No. 8553) supplemented with Protease Inhibitor (cOmplete™, Mini, EDTA-free Protease Inhibitor Cocktail Sigma-Aldrich, Cat. No. 04693159001) and Phosphatase Inhibitor (PhosSTOP, Sigma-Aldrich, Cat. No. 04906837001). Samples were incubated on ice for 10 mins with agitation, samples were then centrifuged at 13,000 RPM for 10 mins and supernatant containing cytosolic proteins was harvested. The pellet was washed with permeabilization buffer and samples were resuspended in RIPA lysis and extraction buffer (ThermoFisher scientific, Cat. No. 89901) with PMSF (Cell Signaling Cat. No. 8553) supplemented with Protease Inhibitor (cOmplete™, Mini, EDTA-free Protease Inhibitor Cocktail Sigma-Aldrich, Cat. No. 04693159001) and Phosphatase Inhibitor (PhosSTOP, Sigma-Aldrich, Cat. No. 04906837001). Samples were incubated on ice with agitation for 10 mins and after centrifugation at 13,000 RPM, supernatant containing the membrane and nuclear soluble fraction was harvested. The pellets were finally washed with solubilization buffer and resuspended using ULTRARIPA kit (Abnova, Cat. No. K6023) supplemented with Halt protease inhibitor (ThermoFisher scientific, Cat. No.78430), Halt Phosphatase inhibitor (ThermoFisher scientific, Cat. No.78428), and PMSF (Cell Signaling Cat. No. 8553), in addition to 1 μl benzonase (Millipore Cat. No. 70746-3). Samples were then sonicated and vortexed and centrifuged at 13,000 RPM for 10 min. The supernatant containing the RIPA insoluble lipid rafts, nuclear insoluble fraction, and bound nuclear chromatin was then treated with NuPAGE™ Sample Buffer (Life technologies Cat. No. NP0007) prior to SDS-PAGE (as described above).

### 2.6 Immunoprecipitation

Protein G Dynabeads (15 µl) (ThermoFisher, Cat. No. 10004D) in PBS with Tween-20 (0.1%) were incubated with 5 µg of goat-anti-human IgG-Fc (ThermoFisher Cat. No. 31125; anti-human IgG-Fc binds the Fc domain of Selicrelumab and HERA-CD40L) for 2 h with rotation at 4°C. After binding of antibodies the beads were washed 2x with PBS containing Tween-20 (0.1%). iDCs were harvested 7 days after differentiation from isolated monocytes (as described above and stimulation with the indicated treatments (500 ng/ml respectively). Stimulation was halted with ice cold PBS and washed 2x in ice cold PBS. Cells were lysed for 30 mins on ice without agitation with Cell Lysis Buffer (Cell signaling, Cat. No. 9803) supplemented with PMSF (Cell Signaling Cat. No. 8553), Protease Inhibitor (cOmplete™, Mini, EDTA-free Protease Inhibitor Cocktail Sigma-Aldrich, Cat. No. 04693159001) and Phosphatase Inhibitor (PhosSTOP, Sigma-Aldrich, Cat. No. 04906837001). For negative controls, Selicrelumab or HERA-CD40L were added to the iDC lysate from non-stimulated cells. After 15 min incubation on ice the lysates were used for immunoprecipitation (IP). Samples were centrifuged at 13,000 RPM. Lysates were incubated with the prepared Dynabeads for 90 mins with rotation at 4°C. After IP, the supernatant was removed and the beads were washed 5x with lysis buffer. Protein complexes were released from the beads with 15 µl of 0.1 M Glycine buffer (pH 2.7) and subsequently with 15 µl of 2x SDS sample buffer. Samples were then processed for SDS-PAGE and western blot analysis (as described above). In some experiments, the lysates after 1^st^ IP (post IP) were used for a 2^nd^ CD40 IP. In this case, the Dynabeads were incubated with a commercially available mouse anti human CD40 antibody (clone G28.5, Bio X Cell, Cat No. BE0189R005MG).

IP of TRAF2 and TRAF6: Protein G Dynabeads were incubated with mouse-anti-human TRAF2 (R&D Systems Cat. No. MAB3277) or mouse-anti-human TRAF6 (R&D Systems Cat. No. MAB3284-100). In order to inhibit the binding of the Fc part of Selicrelumab or HERA-CD40L to the Protein G Dynabeads, 5 µg of goat-anti-human IgG-F(ab)_2_ fragment (JacksonImmunoResearch Cat. No. 109-006-008) was added to the lysates of non-stimulated and Selicrelumab/HERA-CD40L stimulated iDCs.

### 2.7 Immature Dendritic cell intracellular and surface FACS analysis

iDCs were harvested 7 days after differentiation from isolated monocytes (as described above) and stimulation with the indicated treatments (100 ng/ml respectively). Stimulation was halted with ice cold PBS with 2% PFA for 20 mins for fixation and washed 2x in ice cold PBS. Cells were permeabilized with PBS with 0.1% Saponin, 5% FBS and human IgG (Gammunex-C, 10% IgG solution) or ice-cold methanol (80%) (for intracellular phosphoproteins) for 30 mins. For the analysis of cytokine expression, the iDCs were stimulated in presence of Monensin (Biolegend Cat. No. 420701). Samples were washed 2x in PBS with 5% FBS. Cells were then incubated with the indicated detection antibodies in washing buffer and measured with the Guava^®^ easyCyte™ Flow Cytometer for analysis in Flowjo (version 10).

### 2.8 Macrophage functional analysis

Human macrophages (M1/M2a/M2d) were stimulated with the indicated treatments (100 ng/ml respectively on day 0, 3, and 6) and were harvested on day 7 after differentiation from isolated monocytes (as described above). For surface marker analysis, macrophages were harvested and directly measured using BD FACS Celesta™ Cell Analyzer for analysis in Flowjo (version 10). For cytokine analysis, macrophages were treated with 10 ng/ml LPS on day 7 overnight and supernatants were harvested and measured using LEGENDPlex multiplex assay (BD Biosciences, Cat. No. 740509). For phagocytosis analysis, Jurkat cells were cultured in RPMI 1640 medium supplemented with 5% FBS, penicillin (100 U/ml) and streptomycin (100 μg/ml) and first stained with Dil Stain (1,1’-Dioctadecyl-3,3,3’,3’-Tetramethylindocarbocyanine Perchlorate (‘DiI’; DiIC_18_ (3))) (Invitrogen, Cat. No. 11580276) prior to co-culturing with differentiated macrophages (M1/M2d) on day 7 for 4 hours. Macrophages were then measured for Dil positivity using BD FACS Celesta™ Cell Analyzer for analysis in Flowjo (version 10).

### 2.9 *In vivo* CT26 mouse model

For CT26 mice, *in vivo* mouse experiments were undertaken by Heidelberg Pharma (Schriesheimer Str. 101, D-68526 Ladenburg). In short, 7 weeks old female BALB/c mice were subcutaneously inoculated with 5x10^5^ CT26 WT tumor cells into their right flanks. Animals were selected 14 days later according to tumor volume (mean tumor volume 106.66 mm^3^) (day 0). Since human CD40L does not bind to murine CD40, mice received either the fully murine protein acting as a surrogate for the fully human HERA-CD40L (denoted as mmHERA-CD40L) (10 mg/kg) or PBS by intravenous (tail vein) injection on days 0, 3, 7 and 11. Tumor volume and body weight were determined three times per week before group allocation and twice per week afterwards. Tumor and spleen samples were collected from each animal on the day of sacrifice and analyzed by flow cytometry using BD FACS Celesta™ Cell Analyzer for analysis in Flowjo (version 10), and percentage of infiltrating CD8^+^ were calculated from total intratumoral cells. Tumor volume was measured on days as indicated by caliper measurement and size was calculated according to the equation: volume = W^2^ x L x 0.5 (L= length and W= perpendicular width of the tumor, L>W).

### 2.10 *In vivo* TRAMP-C1 mouse model

The TRAMP-C1 prostate carcinoma cells were purchased from ATCC and maintained in DMEM high glucose medium, supplemented with 4 mM L-glutamine, 5% FBS, 5% Nu Serum (Corning, Bedford), 0.005 mg/mL of bovine insulin, and 10 nM dehydroisoandrosterone (Sigma, UK). All animal experiments using the TRAMP-C1 tumor model were performed under United Kingdom Home Office Licenses held at the CRUK Manchester Institute, University of Manchester (PCC943F76). Prior to *in vivo* experiment, cells were screened for mycoplasma contamination and mouse hepatitis virus (MHV) at the Molecular Biology Core Facility (CRUK Manchester Institute). Mice were housed on a 12/12 light/dark cycle and were given filtered water and fed *ad libitum*. C57BL/6 male mice (5-6 week old) were purchased from Envigo, UK. At 6-8 weeks old, male mice were subcutaneously injected with TRAMP-C1 cells (5×10^6^/mouse) and tumor volume was measured *via* a caliper and calculated by using the formula W^2^ x L x 0.5 (L= length and W= perpendicular width of the tumor, L>W). The tumors were established approximately 5-6 weeks post implant and tumor bearing mice were randomized to treatment group once the mean tumor volume was at around 100 mm^3^.

**Figure 1:**
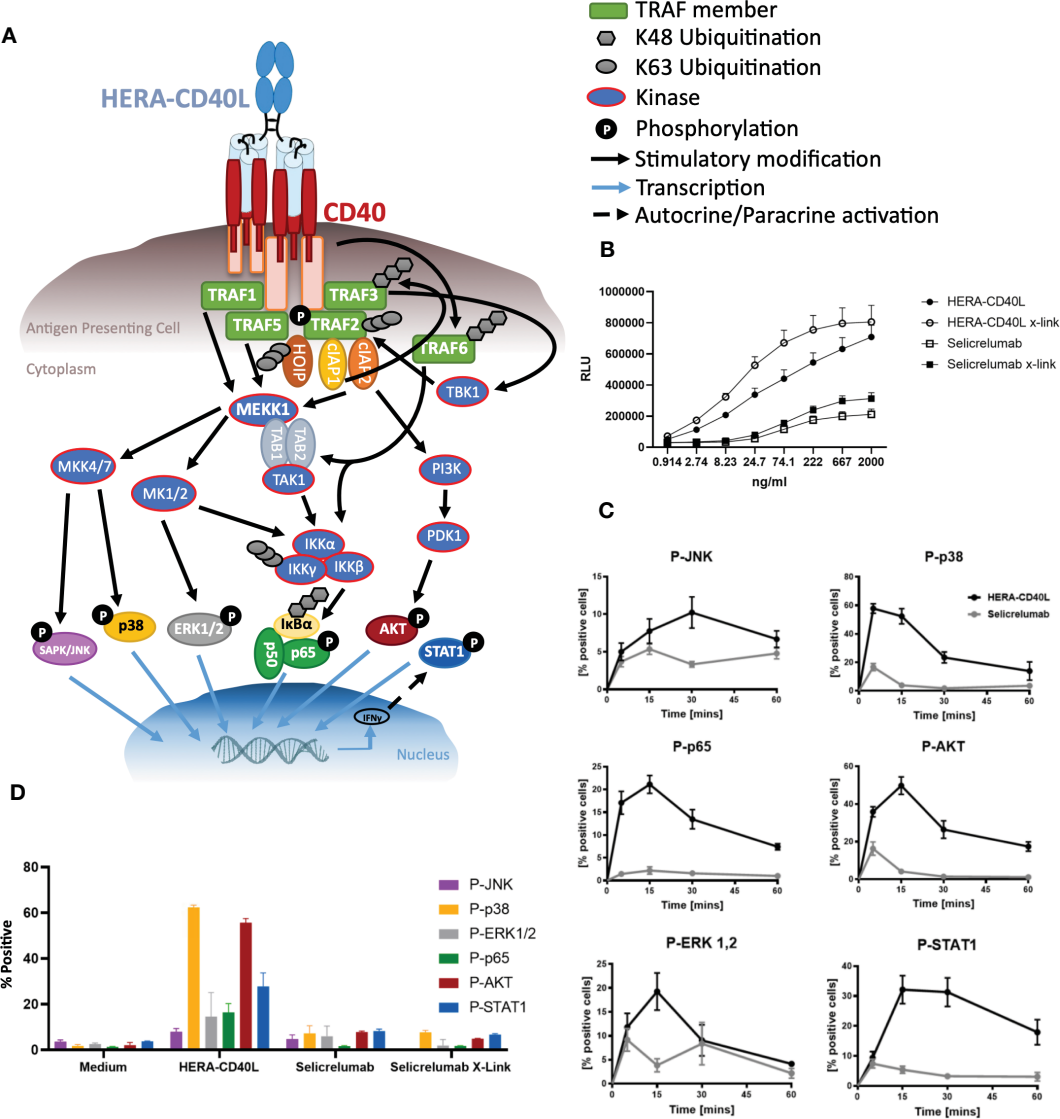
Analysis of HERA-CD40L or Selicrelumab binding to CD40 and activation of signal transduction. **(A)** Schematic overview of the CD40 signaling pathway and related adaptor proteins involved in signal transduction leading to transcription factor activation. **(B)** CD40 luciferase assay comparing HERA-CD40L and Selicrelumab in activating CD40. NFκB-luc2/CD40-expressing Jurkat cells were plated and incubated at 37°C prior to addition of the indicated concentrations of HERA-CD40L or Selicrelumab. The luciferase assay reagent was added and luminescence (RLU) was measured, data displayed as mean + SEM (n=3). Selicrelumab X-link indicates cross-linking of Selicrelumab *via* its Fc domain using an equal amount of IgG Fc Dianova anti-Fc antibody (309-005-008) **(B, D)**. **(C, D).** Inflammatory activation of immature dendritic cells. Monocytes isolated from PBMCs were differentiated to immature dendritic cells and treated with either HERA-CD40L or Selicrelumab and the activation of key indicated inflammatory/survival pathway and transcription factors were measured *via* FACS analysis over a period of 60 mins **(C)**. **(D)** Representation of the effects on inflammatory responses after 15 mins. Colors of the bars match with the colors of the according phosphorylated marker proteins depicted in **(A)**. Data displayed as mean +/- SEM (n=3-5) **(C, D)**.

Irradiation was performed using our previously described method (28) or using the setup as described there forth. In brief, tumor bearing mice were placed in a lead jig and shielded with just the tumors exposed. Mice were treated with X-ray (XSTRAHL, UK) top down operating at 50 KV, 10 mA with a 0.57 AL filter which gave a dose rate of 1.15 Gy per minute. Dosimetry for X-Ray was performed by the Christie Hospital Medical Physics regularly to ensure correct dose rate was delivered to the tumor. Radiotherapy was administered with a total dose of 24 Gy delivered over 3 fractions of 8 Gy over 3 consecutive days (0, 1, 2, respectively). mmHERA-CD40L was administered intraperitoneally on days 2 (200 µg), 5 (100 µg), 9 (100 µg) and 12 (100 µg) per mouse. Anti-PD-1 (clone RMP1-14; BioXCell, USA) antibody was administered at 10mg/kg, 3 times per week over 2 week period as per the schema (**Figure 1A**). A cohort of mice were culled on day 10 (after 3^rd^ dose of mmHERA-CD40L), and the tumor sample harvested and cut into half for ex vivo analysis using IHC and/or for flow cytometry.

### 2.11 Immunohistochemistry

Immunohistochemisty for CT26 tumor samples was performed both, manually on the bench or on automated staining platform. For immunohistochemistry (IHC), tissue samples were fixed in 4% buffered formaldehyde and embedded in paraffin. Sections were cut to 5 μm thickness and dewaxed in xylol for 10 mins (3x), 100% ethanol for 5 mins (2x), 96% ethanol for 5 mins, 90% ethanol for 5 mins, 70% ethanol for 5 mins, then washed with deionized H_2_O for 1 min and then 5 mins. Antigen demasking is dependent on the primary antibody. Target retrieval solution pH 6.0 (DAKO Cat. No. S1699) was used for F4/80 antibody and target retrieval solution pH 9.0 (DAKO Cat. No. S2367) for CD8, CD163 and CD40 antibodies. After cooling, samples were washed in PBS and blocked with blocking solution (Zytomed, Cat. No. ZUC007-100). After washing in PBS, samples were incubated with the indicated primary antibody diluted in antibody diluent buffer (Zytomed, Cat. No. ZUC025-100) for 60 mins at room temperature. For detection, anti-rat polymer-AP (Zytomed Cat. No. RT518H) and anti-rabbit polymer-AP (Zytomed Cat. No. ZUC031-006) were used and Liquid permanent red (DAKO Cat. No. K0640) was employed as substrate for alkaline phosphatase (AP). Samples were imaged using a Keyence BZ-9000E Inverted fluorescence phase-contrast microscope.

For TRAMP-C1 tumor samples, the slides were deparaffinized in xylene followed by rehydration in ethanol. Antigen retrieval was performed using pH 6 citrate buffer, followed by 3% H_2_O_2_ block. Slides were incubated with 10% serum, prior to incubation with primary antibody overnight at 4°C (**Supplementary Table S1**). Primary antibody was detected with either HRP detection kit or biotinylated secondary antibody followed by ABC detection kit (Vector Labs, Burlingame, CA, USA). Slides were briefly incubated in DAB substrate (Vector Labs), washed in water, and counter-stained using haematoxylin. For multiplex staining of mouse FFPE tumor sections, the opal TSA detection system (Opal 520^®^, 570^®^ and 650^®^) were applied to sections following manufacturer’s instruction and run on Leica BOND Rx automated system.

### 2.12 Flow cytometry for ex vivo tumor samples

For flow cytometry analysis, tumors were dissociated with a gentle Macs Dissociator and a murine dissociation kit (Miltenyi Biotec, 130-096-730), as per manufacturing instructions. The dissociated cells were washed with 1% FBS in PBS buffer, and incubated on ice for 30 minutes with anti-CD16/CD32 Fc (ThermoFisher) to block any unspecific binding. Afterwards, cells were incubated in a master mix solution of 1% FBS in PBS buffer containing anti-CD45, anti-CD11b, anti-MHCII, anti-CD80, anti-CD86, anti-CD163 and anti-CD206. For analysis, live cells were gated using vital dye exclusion (L34963, ThermoFisher), and population phenotyped on Fortessa (BD) and analyzed *via* FlowJo software (version 10). Flow cytometry analysis for macrophages was performed on live single cells gated as CD45+CD11b+ cells, followed by the gating on each of the specific markers (i.e.: CD80, MHCII, CD86, CD206, CD163). M1 macrophages were defined as CD45+CD11b+CD80+ cells and as CD45+CD11b+MHCII+. M2 macrophages were defined as CD45+CD11b+CD86+, CD45+CD11b+CD206+, CD45+CD11b+CD163+ cells. Gates were determined by FMO controls for each specific marker.

### 2.13 Image acquisition and analysis

All chromagen slides were scanned digitally using Leica SCN-4000 slide scanner (Leica Microsystems, Germany), and the multiplex slides were scanned using either the Versa Slide scanner (Leica MicroBiosystems, UK) or the VS-120 slide scanner (Olympus). Image analysis and quantification was performed using Halo^®^ image analysis (Indica Labs^®^). Quantification of the positive cells was determined using either haematoxylin or DAPI staining to identify total cell nuclei. CT26 sample slides were imaged using a Keyance BZ-X800 compact microscope.

### 2.14 Statistical analysis

Statistics were calculated using GraphPad Prism (version 9.0) using standard T-test, one-way or two-way ANOVA. Statistics are represented as *p-*value = <0.05 (*), *p-*value = <0.005 (**), *p-*value = <0.0005 (***), and *p-*value = <0.0001 (****). To evaluated the efficacy from the combination studies, Log-Rank Mantel–Cox tests were performed on Kaplan–Meier plots. Survival plot was calculated for mice alive at day 21 with cut off at 300-350mm^3^.

## 3 Results

### 3.1 HERA-CD40L rapidly activates a multitude of inflammatory/survival pathway and transcription factors in human dendritic cells

In order to investigate the impact of HERA-ligands on TNFRSF agonism, we first analyzed the level of potential activation using an artificial luciferase system (recombinant Jurkat cells). HERA-CD40L was compared in its capacity to induce CD40 mediated luciferase expression to Selicrelumab, a well-characterised therapeutic antibody targeting CD40 (29) (**Figure 1B**). Using a dose-response approach, comparing Fc-mediated cross-linked versus non cross-linked treatments, HERA-CD40L demonstrated a marked increase in CD40 activation despite a clear but less pronounced activation being induced by Selicrelumab. Selicrelumab has been reported to exert activity without secondary crosslinking *via* Fc-receptors (30, 31), and indeed, cross-linking Selicrelumab *via* Fc-binding antibodies enhanced CD40 activation only slightly, similar to the slightly increased activation by cross-linked HERA-CD40L. However, the Selicrelumab induced activity can be mainly attributed to the aggregates in this protein batch as shown by luciferase activity determinations of the respective size exclusion chromatography (SEC) fractions (**Supplementary Figure 1A**). Interestingly, Selicrelumab counterintuitively showed a stronger binding to immature dendritic cells than HERA-CD40L (see **Supplementary Figure 1B**). In order to determine the relative downstream effects of the increased activation of CD40 signaling according to luciferase activity, monocytes were isolated and differentiated to immature dendritic cells (iDCs). iDCs were then treated with either HERA-CD40L or Selicrelumab and the phosphorylation of inflammatory/survival pathway and transcription factors (SAPK/JNK, p38, ERK1/2, NFκB (p65/Rel-A), AKT, and STAT1; for an overview see **Figure 1A**) was measured *via* FACS analysis. It was determined that under all conditions, HERA-CD40L induced significant levels of inflammatory/survival pathway and transcription factor phosphorylation in a time dependent manner (peaking between 15-30 mins post treatment), while Selicrelumab induced only very little activity – even if it is cross-linked *via* the Fc region (**Figures 1C, D**).

### 3.2 HERA-CD40L induces a loss of TRAF3 and cIAP1, leading to phosphorylation of TRAF2.

**Figure 2:**
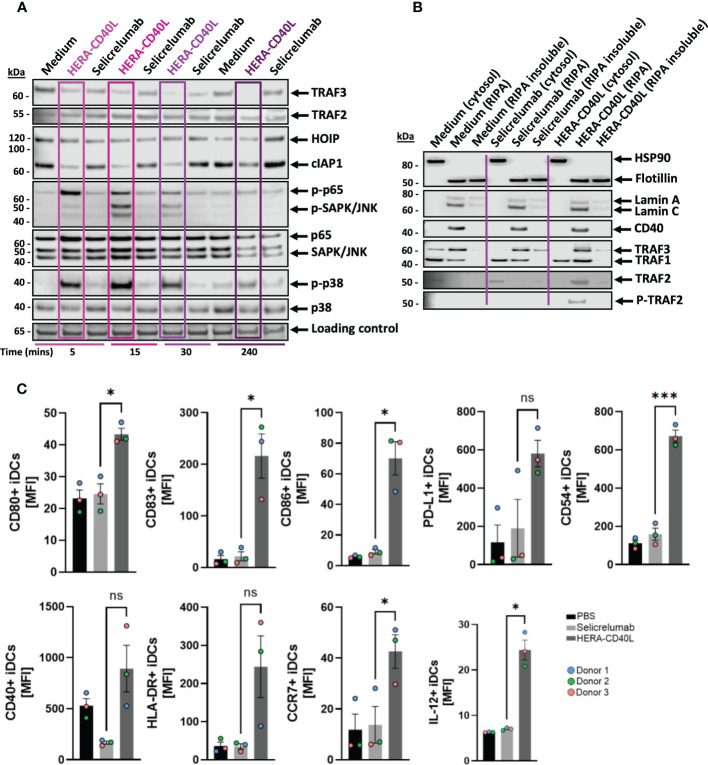
Analysis of the activity of CD40 related proteins involved in transduction leading to activation of key inflammatory/survival pathway and transcription factors. **(A, B)** Immature dendritic cells were differentiated from monocytes derived from PBMCs donors and treated with the indicated treatments over 4 hours. **(A)** Representative western blot analysis (*n=3-6*) of CD40 related signaling adaptor proteins and related inflammatory/survival pathway and transcription factors using beta-actin as a loading control. HERA-CD40L treatment is highlighted by purple rectangles shaded by timepoints. **(B)** Representative subcellular fractionation and western blot analysis 15 minutes after treatment (n=3), the indicated subcellular fractions are determined by cytosolic, RIPA soluble and insoluble samples, as indicated. RIPA samples correspond to membrane and nuclear fractions while RIPA insoluble samples correspond to insoluble lipid rafts, nuclear insoluble fraction, and bound nuclear chromatin. **(C)** Dendritic cell activation surface marker expression analysis and IL-12 production comparison between the indicated treatments. Data displayed as mean +/- SEM (n=3 donors), Standard unpaired t-test comparison. Statistics are represented as either not significant (ns), p-value = <0.05 (*), or p-value = <0.005 (***).

To determine why Selicrelumab induces less downstream signal transduction activation of CD40 in comparison to HERA-CD40L despite both displaying an ability to bind, protein members of the CD40 signaling complex and downstream signaling were analyzed *via* western blot. For TRAF3, an adaptor protein of the activated CD40 receptor (32, 33), it was observed that for HERA-CD40L, a clear degradation occurred until no more protein was detected at 4 hours post-treatment. This correlated with activation of NFkB non-canonical pathway and processing of p52 (**Supplementary Figure 2A**). For Selicrelumab, a loss of TRAF3 was observed after 30 mins, however levels returned to normal at 4 hours (**Figure 2A**). In contrast to TRAF3, which is degraded, TRAF1 is upregulated after stimulation with HERA-CD40L (**Supplementary Figure 2A**). For TRAF2, no difference was observed in the steady-state levels (**Figure 2A**). For HOIP (RNF31), an E3 ligase facilitating downstream signaling (34), a small decrease in expression was observed for HERA-CD40L and Selicrelumab in the first 5-15 mins. While Selicrelumab returns to baseline expression at 30 mins post treatment, the decrease in expression was sustained by HERA-CD40L. For cIAP1, upon CD40 receptor-ligand interaction, it forms a complex with TRAF2/3 and directly ubiquitinates these proteins *via* the BIR1 domain - a conserved structure within the IAP family (35–37). The BIR3 domain of cIAP1 regulates its self-ubiquitination due to engagement with smac, inducing its own rapid degradation (36, 38). The degradation of cIAP1 was clear for HERA-CD40L, while no strong degradation was seen for Selicrelumab treatment, and this signal correlated with the degradation of TRAF3, which is known to be a negative regulator of CD40 signaling on B cells (39) (**Figure 2A**). Indeed, when phosphorylation levels of NFκB (p65), SAPK, and p38 were measured, the degradation of TRAF3 and cIAP1 correlated in a time dependent manner with the induction of phosphorylation (**Figure 2A**), this was further highlighted for NFκB (p65) and SAPK in subcellular fractionation analysis (**Supplementary Figure 1C**). In contrast to Selicrelumab, translocation of Phospho-p65 to the nucleus has been demonstrated after stimulation with HERA-CD40L. Further, no phosphorylation of these inflammatory/survival pathway and transcription factors was detected upon Selicrelumab treatment, consistent with FACS analysis shown above. To further determine the mechanism behind HERA-CD40L mediated CD40 activation, a subcellular fractionation assay was used to assess the relative levels of receptor adaptor proteins (TRAF family members). Interestingly, TRAF1 and TRAF3 were detected in the membrane fractions for both HERA-CD40L and Selicrelumab treatment, however only with HERA-CD40L treatment did we detect phosphorylation of TRAF2 (40) (**Figure 2B**). To compound these observations, we next determined the translational effects of the enriched transcription factor activation of HERA-CD40L compared to Selicrelumab. iDCs were measured *via* FACS analysis of key surface markers (CD80, CD83, CD86, CD54, HLA-DR, CCR7) demonstrating a pro-inflammatory phenotype (41), in addition to increased levels of IL-12 production (**Figure 2C**) for cells treated with HERA-CD40L. In contrast, treatment with Selicrelumab did not alter these key surface markers and did not change IL-12 production.

### 3.3 Selicrelumab fails to induce strong phosphorylation of TRAF2 in comparison to HERA-CD40L

**Figure 3:**
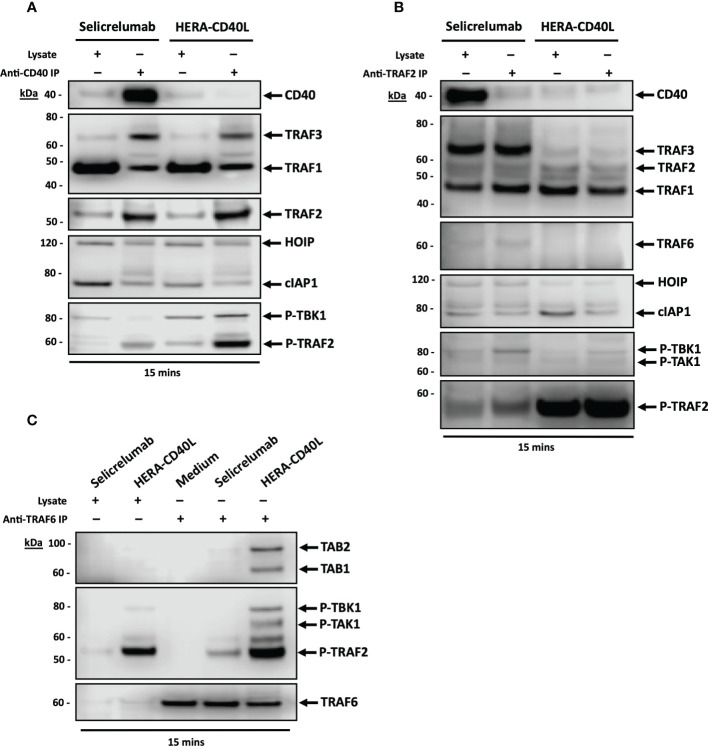
Immunoprecipitation analysis of CD40 and associated proteins involved in signal transduction 15 mins post treatment. **(A–C)** Representative Immunoprecipitation and western blot analysis of immature dendritic cells differentiated from monocytes derived from PBMCs. Lysates were incubated with beads coated with the either CD40 **(A)**, TRAF2 **(B)**, or TRAF6 **(C)** antibodies, the indicated bound proteins to the respective target were detected *via* SDS-PAGE and then western blot analysis (n=3).

To confirm the importance of the receptor adapter proteins in the formation of a signaling complex leading to inflammatory/survival pathway and transcription factor activation, immunoprecipitation of CD40, TRAF2, and TRAF6 was carried out. Much more CD40 was immunoprecipitated with anti-human IgG-Fc coated beads after Selicrelumab stimulation of iDCs compared to HERA-CD40L stimulation. However, the signaling complex after HERA-CD40L stimulation induces a stronger signal transduction than that observed for Selicrelumab. For immunoprecipitation of CD40 (**Figure 3A**), the levels of TRAF1, TRAF2, TRAF3, HOIP were comparable for the treatments with Selicrelumab and HERA-CD40L, but for cIAP1, levels were lower for HERA-CD40L and consistent with the previously described results in **Figure 2**. In contrast to Selicrelumab, stimulation of iDCs with HERA-CD40L leads to recruitment and phosphorylation of TBK1 to the signaling complex and TBK1 mediated phosphorylation of TRAF2. No phosphorylation of TBK1 and much weaker phosphorylation of TRAF2 was observed for Selicrelumab, while a strong signal was seen for HERA-CD40L treatment. Only a part of the CD40 molecules are involved in formation of a signaling complex after stimulation of iDCs with Selicrelumab or HERA-CD40L (1^st^ immunoprecipitation, IP). Higher amounts of CD40 are immunoprecipitated in a 2^nd^ IP with mouse-anti-human CD40 Ab. However, no or much lower amounts of proteins involved in signal transduction are bound to CD40 in the 2^nd^ IP (**Supplementary Figure 2B**). Immunoprecipitation of TRAF2, which was not recruited to the signaling complex, showed higher binding of TRAF1 and p-TBK1 but lower phosphorylation of TRAF2 after stimulation with Selicrelumab compared to HERA-CD40L (**Figure 3B**). We did not find any TRAF6 in the signaling complex after stimulation of iDCs (data not shown). However, immunoprecipitation of TRAF6 after stimulation with HERA-CD40L led to recruitment of TAB1, TAB2, p-TAK1 and also p-TBK1 and p-TRAF2 in contrast to Selicrelumab (**Figure 3C**). Thus, taken together, it appears that a signaling complex is formed upon HERA-CD40L treatment that results in a strong phosphorylation of TRAF2, an event that is not seen for Selicrelumab binding to CD40, suggesting a pivotal step in the receptor mediated signal transduction for CD40 agonism, and that downstream signaling is reflective of the level of TRAF2 phosphorylation and recruitment and activation of TAK1 to TRAF6.

### 3.4 HERA-CD40L repolarizes tumor associated macrophages into a pro-inflammatory state

**Figure 4:**
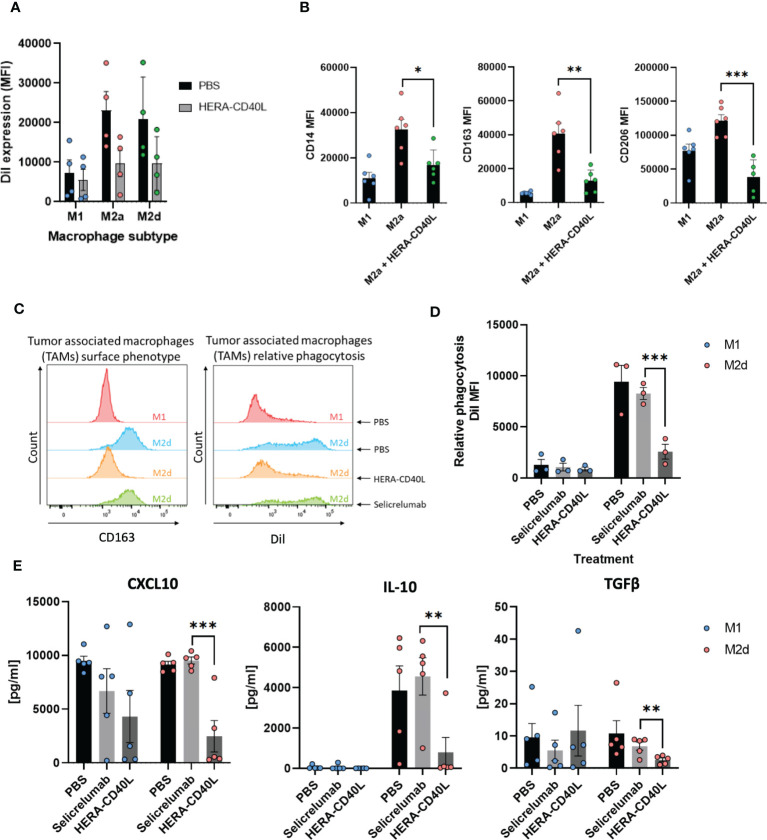
Differentiation of monocytes to macrophages and analysis of polarization states. **(A, C)** Macrophages were differentiated from monocytes derived from PBMCs and polarized to either M1 or M2 macrophages (subtypes of M2a or M2d were generated, depending on the assay). **(A)** Comparison of macrophage subtypes. M1, M2a, and M2d macrophages were generated and treated with either PBS or HERA-CD40L (n=4 donors) to compare responses of each subtype to HERA-CD40L using relative phagocytosis. **(B)** M1 and M2a macrophages were generated and expression of defined M2 surface markers were analyzed and compared post-treatment with HERA-CD40L. **(C)** Representative FACS histogram plot of the expression profile of CD163, a known M2 expression marker, congruent with the phagocytosis functional indicator (Dil stain) of TAMs (M2d macrophages) compared to M1 macrophages. **(D)** Relative phagocytosis after treatment of M1 and M2d macrophages with Selicrelumab or HERA-CD40L (n=3 donors). **(E)** Detection and quantification of the indicated suppressor associated cytokines comparing M1 macrophages to M2d Macrophages (n=5 donors). Data displayed as mean +/- SEM **(A, C)**. Standard unpaired *t*-test comparison. Statistics are represented as either p-value = <0.05 (*), p-value = <0.005 (**), or p-value = <0.0005 (***).

Previous data has shown that anti-inflammatory markers are reduced while pro-inflammatory markers are increased in macrophages following HERA-CD40L treatment, suggesting a repolarization effect (23). We examined M2a, and in addition, M2d macrophages, which are closely related to tumor-associated macrophages (TAMs), in their functional response to HERA-CD40L. Both M2a and M2d macrophages demonstrated a high phagocytosis response to Jurkat tumor cells, a known trait for anti-inflammatory macrophages, which was equally reduced upon HERA-CD40L, suggesting M2a and M2d macrophages in this context are comparable (**Figure 4A**) (42). Upon treatment with HERA-CD40L, the surface marker expression of CD14, CD163, and CD206 of M2a macrophages was significantly reduced to M1-like levels (**Figure 4B**). This surface phenotype and the capacity for macrophages to induce phagocytosis was also markedly reduced to more M1-like levels compared to control in M2d macrophages (**Figures 4C, D**). In contrast, the addition of Selicrelumab did not alter phagocytotic activities. Further analysis of IL-10, CXCL10, and TGFβ (pro-tumor secreted markers) highlighted the functional repolarization of HERA-CD40L treated M2d macrophages, whereas Selicrelumab had no effect (**Figure 4E**). In an allogeneic co-culture of immature dendritic cells and T cells, we observed an increase in CD45RO^+^, CD54^+^, CD69^+^, and CD25^+^ T cells, a proportionate decrease in CD45RA^+^ T cells, and an increase in the secreted pro-inflammatory markers TNFα, IFNγ and IL-2 upon treatment with HERA-CD40L (**Supplementary Figure 3**). In contrast to Selicrelumab stimulation, naïve CD4 T lymphocytes are clearly more potently stimulated in co-culture with HERA-CD40L stimulated iDCs leading to polarization to Th1 lymphocytes as demonstrated by IFNγ production. These results suggest an induction of T cell responses upon CD40 stimulation of immature dendritic cells by HERA-CD40L. This trend on T cell responses was also demonstrated in intratumoral CD8^+^ T cells in a pmel-1 T cell receptor transgenic mouse model (**Supplementary Figure 4**), where cytotoxic CD8^+^CD107a^hi^ were increased in the spleen and tumor upon treatment with HERA-CD40L, while additionally reducing tumor growth by up to 69%.

### 3.5 HERA-CD40L induces an anti-tumor response *in vivo*

**Figure 5:**
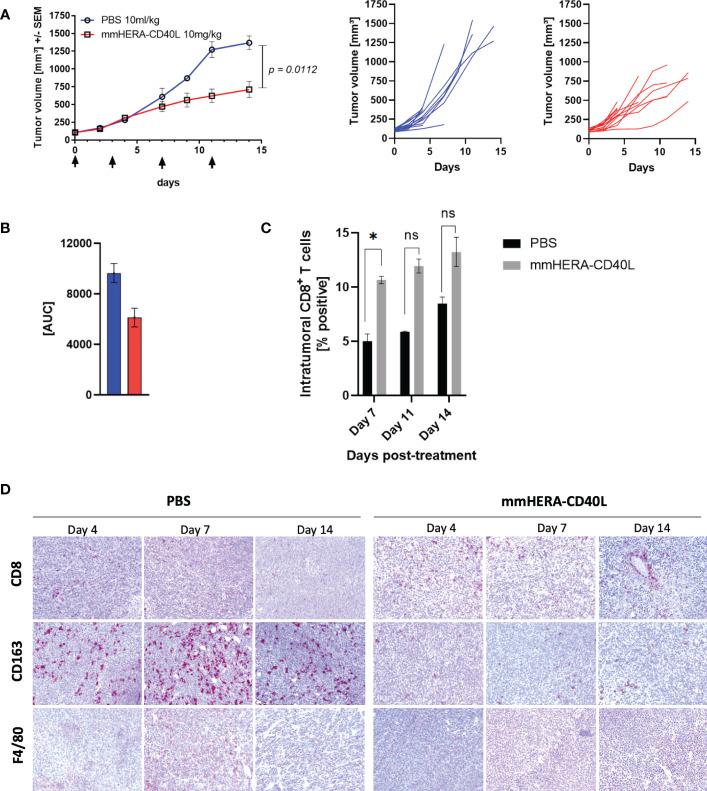
*In vivo* CT26 mouse study comparing the effect of murine HERA-CD40L (mmHERA-CD40L) on tumor growth and the translational effect on the TME. **(A)** Tumor growth inhibition as measured by mean tumor volume (MTV) of CT26 tumor bearing BALB/c mice treated with mmHERA-CD40L (10mg/kg) x4 at the indicated days and associated spaghetti plot. Data displayed as mean +/- SEM (n=6-12). **(B)** Calculation of area under the curve (AUC) for PBS (10ml/kg) versus mmHERA-CD40L (10mg/kg) (as mean +/- SEM). **(C)** FACS analysis of intratumoral CD8^+^ T cells at 7, 11, and 14 days post-treatment comparing PBS (10ml/kg) and mmHERA-CD40L (10mg/kg). Data displayed as mean +/- SEM. **(D)** Representative images of immunohistochemistry analysis of the indicated expression markers (n=3 mice each). Standard unpaired *T*-test comparison. Statistics are represented p-value = <0.05 (*) or exact p-value. ns, not significant.

Next, we investigated anti-tumor therapy of mmHERA-CD40L using CT26 bearing mice, which were treated 4x with either PBS (10ml/kg) or mmHERA-CD40L (10mg/kg). Since human CD40L does not bind to mouse CD40 (43), the murine surrogate mmHERA-CD40L was employed for syngeneic mouse tumor studies. Similar *in vitro* activity of human HERA-CD40L and its mouse surrogate mmHERA-CD40L has been shown in a Luciferase reporter gene assay employing human cells which express human CD40 (mouse CD40L binds both, human and mouse CD40) (44), see **Supplementary Figure 1D**. The mean tumor volume of mmHERA-CD40L treated mice was reduced significantly by 48% by day 14 (**Figure 5A**). mmHERA-CD40L was well tolerated with no signs of toxicity, no abnormalities detected at necropsy and no impact on body weight gain of the mice (**Supplementary Figure 5**). Infiltration of CD8^+^ T cells was measured over 3 days (day 7, 11, and 14). On day 7, the % of intratumoral CD8^+^ T cells was 2-fold higher in the mmHERA-CD40L treated mice, correlating with clearly decreased tumor growth measured on day 7 onwards (**Figure 5C**).This was further highlighted upon IHC analysis of tumor sections. We observed a similar increase in CD8^+^ T cells upon mmHERA-CD40L treatment and critically, we also observed a large increase in overall macrophages (as determined by F4/80 expression), while simultaneously reducing the expression of anti-inflammatory marker CD163, further highlighting the effect mmHERA-CD40L has on TAMs. These results corroborate previous pilot *in vivo* experiments where mmHERA-CD40L reduced tumor volume of CT26 mice by 40% and an increase in F4/80 and CD8+ T cells together with a decrease in CD163 in the tumor was demonstrated (**Supplemental Figure 6**). Further, there was a reduction in tumor volume of almost 70% comparing transplanted transgenic pmel-1 CD8^+^ T cells treated with either control or mmHERA-CD40L. Additionally, a trend was observed where an increase in activation markers of mmHERA-CD40L treated pmel-1 CD8^+^ T cells (CD44, CD107a), in contrast to non-treated pmel-1 CD8^+^ T cells (**Supplementary Figure 4**).

### 3.6 HERA-CD40L induces an anti-tumor response *in vivo* in combination with radiotherapy

**Figure 6:**
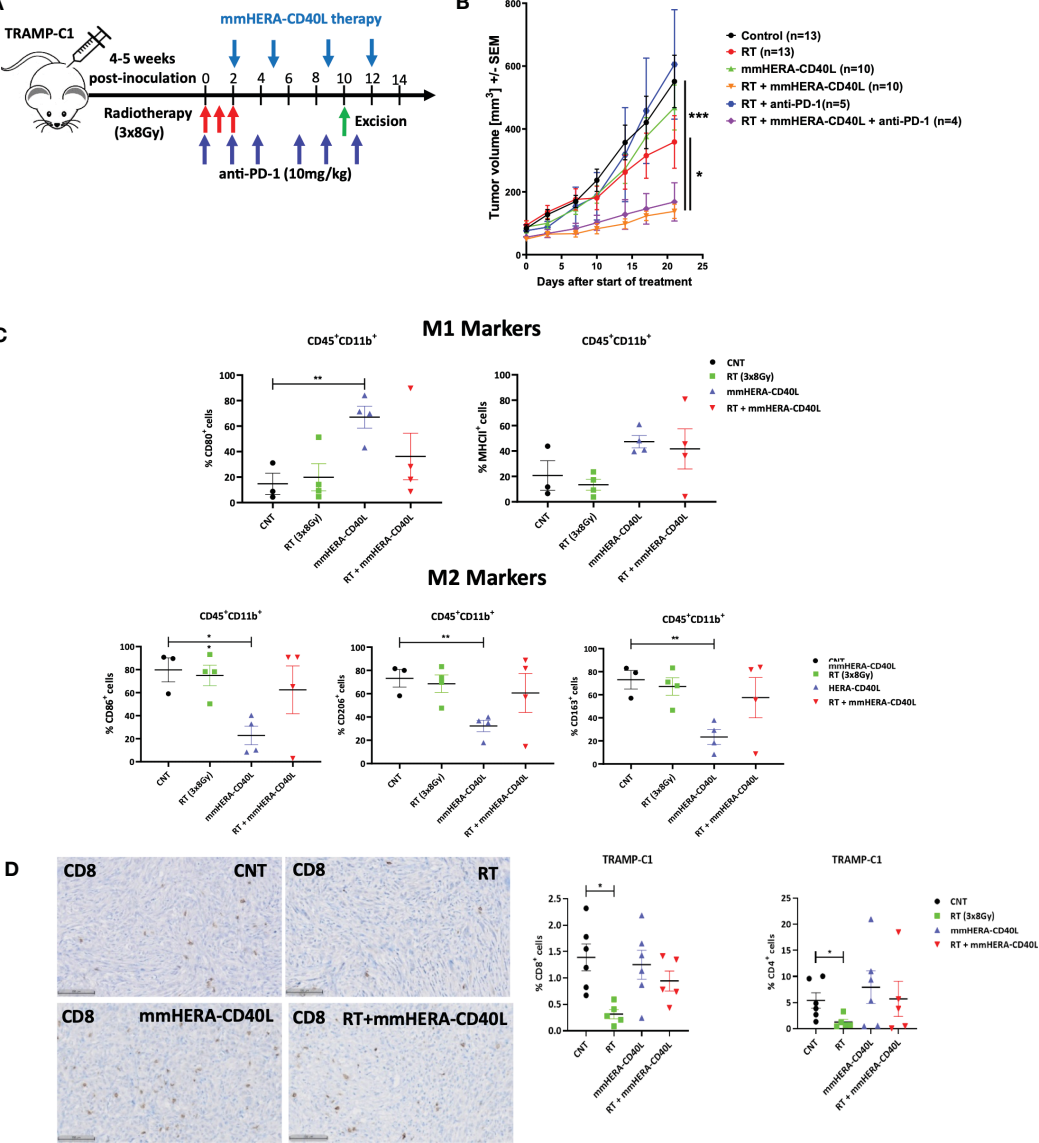
mmHERA-CD40L enhances therapeutic efficacy in combination with RT in the murine prostate TRAMP-C1 tumor model. **(A)** Schematic representation of the treatment schedule. **(B)** TRAMP-C1 tumor growth measured as mean tumor volume (MTV) demonstrating growth delay when combining mmHERA-CD40L with RT. Tumor bearing mice were randomized to treatment group. RT (3x8Gy) was given on day 0, 1, 2. mmHERA-CD40L was administered at 200 µg per mouse on day 2, followed by 100 µg on days 5, 9 and 12. anti-PD-1 was administered at a dose of 10 mg/kg every second day (6 doses in total). A cohort of mice was culled on day 10 and samples were collected for TME characterisation. **(C)** Flow Cytometry of TRAMP-C1 tumors measuring M1 and M2 macrophage specific markers, comparing RT or mmHERA-CD40L alone or in combination. Flow cytometry analysis for macrophages was performed on alive single cells gated as CD45^+^CD11b^+^ cells, followed by the gating on each of the specific markers. **(D)** IHC images and quantification of CD4^+^/CD8^+^ cells within TRAMP-C1 tumors, comparing control and mmHERA-CD40L with RT treated mice - representative images (chromogenic staining) for CD8^+^ in tumor samples. * denotes p ≤ 0.05 (where applicable) as determined by unpaired *t*-test *** denotes p<0.001; ** denotes p<0.01.

Radiotherapy (RT) can induce immunomodulatory changes in both the tumor and the tumor microenvironment (TME). The CT26 syngeneic colorectal tumor models are known to have an increase in TILs relative to other syngeneic models (45) following administration of RT. We have previously demonstrated in the TRAMP-C1 syngeneic tumor model that there is an increase in TAMs and other suppressor cells after treatment with RT, while CD8^+^ T cell, natural killer cell and dendritic cell gene transcripts were reduced post RT (46). Furthermore, we have shown that RT in combination with immune modulatory agents can induce a systemic anti-tumor immune response (47, 48). In this study, we therefore examined the potential for mmHERA-CD40L to be used in combination with radiotherapy using the TRAMP-C1 model. Mice were pre-treated with 3x8Gy RT prior to treatment with mmHERA-CD40L, as depicted in the scheme in **Figure 6A**. No toxicity or significant weight loss was observed in mice which received mmHERA-CD40L compared to control animals. Administration of RT resulted in a transient weight loss when administered alone or in combinations with mmHERA-CD40L and/or anti-PD-1 (**Supplementary Figure 7A**). RT alone or mmHERA-CD40L alone had no significant impact on tumor growth, however, tumor growth inhibition was observed when RT was combined with mmHERA-CD40L (**Figure 6B**). In order establish a potential benefit of adding anti-PD-1 treatment to the combination of mmHERA-CD40L and RT, two further groups with a small number of mice were randomized to treatment groups. The combination of anti-PD-1 monoclonal antibody to RT had no significant impact on the tumor growth compared to RT group alone or the control tumors. Addition of anti-PD-1 therapy concurrently with mmHERA-CD40L and RT to tumor bearing mice resulted in no additional therapeutic benefit compared to animals which received RT and mmHERA-CD40L (**Figure 6B**, **Supplementary Figure 7B**). However, the combination of RT and mmHERA-CD40L showed a significant improvement in survival compared to RT and control mice (**Supplemental Figure 7B**). We then performed flow cytometric analysis in tumor samples which were excised on day 10 as per the schema (**Figure 6A**). As expected, treatment with mmHERA-CD40L led to an increase in M1 markers and a decrease in M2 markers, which was clearly less pronounced in combination with RT (**Figure 6C**, see **Supplemental Figure 8** for further analysis, including PD-1 expression; for gating strategy see **Supplemental Figure 7C**). In line with this finding we observed a loss of intratumoral CD4^+^ and CD8^+^ T cells upon RT, however this was restored upon treatment with mmHERA-CD40L (**Figure 6D**), suggesting that mmHERA-CD40L could counteract the negative immunosuppressive impact of RT and further enhance anti-tumor responses, which is in line previous data depicted in **Figure 4**.

## 4 Discussion

In this manuscript we have investigated the underlying mechanisms behind the response of HERA-CD40L in comparison to Selicrelumab, which has been involved in the completion of three clinical trials (NCT02304393, NCT02665416 and NCT03892525), and is currently involved in three further active trials (NCT03193190, NCT03424005 and NCT03555149), suggesting therapeutic value. We have demonstrated that HERA-CD40L induced a much stronger activation of inflammatory/survival pathways and transcription factors using phospho-specific markers of activation in dendritic cells (**Figure 1C, D**). Key signal transduction pathway members induced by HERA-CD40L mediated activation of CD40 were measured, leading to activation of inflammatory responses. Degradation of TRAF3 4h after stimulation with HERA-CD40L led to accumulation of NIK and activation of non-canonical NFkB pathway (49, 50) and resulted in processing of p52 (**Supplementary Figure 2A**). In contrast to TRAF3, TRAF1, which is unique within the TRAF family as it is the only member to lack a RING domain, is known to be a positive regulator of CD40. TRAF1 is upregulated 4h after stimulation of iDCs with HERA-CD40L (**Supplementary Figure 3A**) and may have regulatory functions at late stage after stimulation (51–54). TRAF1 expression also is reported to be a positive indicator of efficient CD40 activation, which supports the findings that HERA-CD40L induces more efficient activation of CD40 compared to Selicrelumab (55, 56). Recruitment of p-TBK1 to the signaling complex after stimulation of iDCs with HERA-CD40L mediated a strong phosphorylation of TRAF2 on serine 11 (**Figure 3A**), which plays an important role in NFkB and MAPK activation (57). Although we did not find TRAF6 in the signaling complex, IP shows a clear binding of TAB1, TAB2 and recruitment of p-TAK1 to TRAF6 after stimulation with HERA-CD40L in contrast to Selicrelumab (**Figure 3C**). TRAF6 induces activation of TAK1, a MAP3K, to trigger activation of MAPK pathways and transcription factors AP-1 and NFκB (58). TRAF6 can be activated without direct contact to CD40 (59, 60). P-TBK1 and p-TRAF2 in complex with TRAF6 can play a role in activation of TRAF6 after HERA-CD40L stimulation. This is in line with the molecular layout of HERA-CD40L and Selicrelumab. The hexavalent HERA-CD40L comprises of two CD40L trimers and is therefore capable of binding to six CD40 molecules.The CD40L trimers of HERA-CD40L are more closely mimicking physiological agonism that would be achieved through cell to cell interaction, thereby enabling an appropriate assembly of the intracellular CD40 signaling complex. In contrast, the bivalent CD40 binding of Selicrelumab does not result in sufficient CD40 clustering on the surface of the target cell, resulting in a lack of downstream signal transduction activation. Structural details of this concept are illustrated in our previous publication (21). Thus, the level of TRAF2 phosphorylation and recruitment and activation of TAK1 to TRAF6 - resulting in activation of downstream signaling pathways and transcription factors - could act as a marker of effective CD40 activation. Further support for TRAF2 acting as a key component in CD40 signalling has been previously demonstrated in mouse B cells, mouse epithelial cells, and human epithelial cells, such that TRAF2 ubiquitination and degradation following recruitment/phosphorylation was a critical step in CD40 mediated signal transduction (61–64). These effects could not only be seen in dendritic cells, but additionally in other key inflammatory cells, suggesting a highly conserved signaling axis in healthy, functional cells. While immature dendritic cells were driven to be pro-inflammatory by HERA-CD40L as determined by surface marker expression and IL-12 production (41) (**Figure 2C**), macrophages were also repolarized from M2 anti-inflammatory/pro-tumor macrophages (M2a and M2d demonstrated very similar responses, suggesting a high comparability between M2 macrophage subtypes) to pro-inflammatory M1 macrophages in response to HERA-CD40L (42) (**Figure 4**). This effect was not observed with Selicrelumab. Macrophage repolarization could have significant impact on the TME (65) and ultimately have significant impact on the overall efficacy of immunomodulation based therapies, as the TME is highly suppressive to inflammatory responses (66). In line with this, suppressive cytokines were measured and shown to be markedly reduced by TAM-like macrophages when treated with HERA-CD40L compared to Selicrelumab (**Figure 4E**). IL-10 in particular, a regulator of Treg cells, is almost completely lost. Given the synergy of results between the CD40 signaling responses and phenotypes observed for iDCs, TAMs, and T cells, together with supporting literature for TRAF2 as key component for CD40 activation, it is surprising that only a faint CD40 band is seen in the HERA-CD40L immunoprecipitates (**Figure 3A**) which is close to the detection limit of the Western Blot. Our current working hypothesis is that clustering a rather low number of CD40 molecules by HERA-CD40L is sufficient to induce signaling leading to transcription factor activation (**Figure 1C, D**), which has been clearly demonstrated by showing induction of p-TBK1 and p-TRAF2 (**Figure 3A**). Further research on TNFSFRs is needed to clarify this point. Dose-dependent binding of both, Selicrelumab and HERA-CD40L to iDCs has been shown by flow cytometry (see **Supplementary Figure 1B**). Employing the anti-human CD40 antibody (clone G28.5) for a second IP demonstrates, that a high level of CD40 is present, however, the level of signaling relevant proteins (i.e. p-TRAF2) is rather low here (see **Supplementary Figure 2B**, very right lanes). These high levels of CD40 are in line with the subcellular fractionation data shown for HERA-CD40L treatment (RIPA, see **Figure 2B**).

Regarding the impact on the overall immune responses with respect to inflammation and the TME, we explored the efficacy of HERA-CD40L *in vivo*. Using a fully murine surrogate for HERA-CD40L (mmHERA-CD40L) (23), we examined the single agent efficacy of tumor growth inhibition in a CT26 mouse model. We observed a marked reduction in tumor growth of 48% (*p* = 0.0112) (**Figure 5A**). In addition, we observed a significant increase in the number of intratumoral cytotoxic CD8^+^ T cells, up to ~2x fold after 8 days post treatment. Furthermore, the repolarization of TAMs was also demonstrated *in vivo* using IHC - CD163 positive F4/80 Macrophages were lost upon treatment with mmHERA-CD40L, highlighting the pro-inflammatory impact mmHERA-CD40L has on the TME (**Figure 5D**), which is congruent with our *in vitro* data for fully human HERA-CD40L. Macrophage repolarization thus is a point of significant interest for immunotherapy in general, as demonstrated by van Dalen et al. (67) NFkB appears to be a major component in TAM phenotype differentiation, therefore it is likely that HERA-CD40L is having a direct impact on the target macrophages resulting in repolarization from TAM to M1, as supported by the induction of NFkB in **Figures 1**, **2** by HERA-CD40L, though it cannot be ruled out the T cells are also contributing to this effect. As stated previously in the context of checkpoint inhibitors, single agent approaches may not be efficacious enough in the larger proportion of patients. We examined the beneficial role mmHERA-CD40L might have as a combination-based therapy with radiotherapy. Radiotherapy currently plays a pivotal role in the treatment of around half of all cancer patients (68, 69) and more recently the impact of radiotherapy on the TME has gained an increased level of importance over the last decade (70–72). Briefly, radiotherapy has been established in some tumor types to have an immunomodulatory effect, leading to an influx of tumor associated macrophages and myeloid derived suppressive cells, which can be highly immunosuppressive (71–73). We have described the impact HERA-CD40L has on macrophages *in vitro* and *in vivo*, we next analyzed these beneficial properties in a combination-based therapeutic approach using a TRAMP-C1 model. Mice (n=9-12) were treated with either radiotherapy, mmHERA-CD40L, or a combination of both. Neither radiotherapy, nor mmHERA-CD40L alone had a pronounced positive therapeutic impact on tumor growth. In combination however, mice that were pre-treated with 3x8Gy RT and subsequently treated with mmHERA-CD40L had a ~3x fold decrease in tumor growth compared to control mice. Moreover, in line with previously seen intratumoral FACS and IHC analysis, CD4^+^/8^+^ were rescued after radiotherapy by treatment with mmHERA-CD40L. Furthermore, TAMs were decreased by mmHERA-CD40L, while the pro-inflammatory marker CD80 was increased. Together, these results highlight the synergistic effect mmHERA-CD40L treatment has on radiotherapy treatment, providing a balance between RT induced immune suppression and promotion of inflammatory responses by manipulating the TME. In conclusion we have demonstrated that the next-generation TNFRSF agonists, the HERA-ligands, are capable of inducing TNFRSF activation, resulting in enhanced inflammatory response *in vitro* compared to other anti-CD40 agonist mAb such as Selicrelumab, resulting in enhanced tumor control *in vivo*, in combination with RT therapy. Further, combination based approaches with immuno-oncology agents informed by enhanced understanding the mechanisms of targeted approaches, including CD40 agonism, have the potential to further improve cancer outcomes (74–76).

## Data availability statement

The original contributions presented in the study are included in the article/**Supplementary Material**. Further inquiries can be directed to the corresponding authors.

## Ethics statement

The animal study was reviewed and approved by the Ethics Committee for Animal Experimentation. The experimental protocols were registered by the regional board Freiburg (Regierungspräsidium Freiburg; G-15/41) or were performed under a United Kingdom Home Office Licence (PCC943F76) which were reviewed and approved by the CRUK Manchester Institute Animal Welfare and Ethical Review Body (AWERB).

## Author contributions

The manuscript was prepared by JF and MT, **Figures 1** through **5** were prepared by JF, **Figure 6** was prepared by DM, JH, TI, data and statistical analysis was done by JF, DM and ER. Experimental procedures were supervised by JF, KB-F, MS, CM, CG, JS, MT, JH, DM. Mouse experiments and data analysis were prepared by MM, JF, DM, ER, JH, TI. Protein design and production was done by KH, KB-F, and OH. All authors contributed to the article and approved the submitted version.

## References

[1] Schreiber RD Old LJ Smyth MJ. Cancer immunoediting: integrating immunity’s roles in cancer suppression and promotion. Sci (New York NY). (2011) 331(6024):1565–70. doi: 10.1126/science.1203486 21436444

[2] Pan C Liu H Robins E Song W Liu D Li Z. Next-generation immuno-oncology agents: current momentum shifts in cancer immunotherapy. J Hematol Oncol (2020) 13(1):29. doi: 10.1186/s13045-020-00862-w 32245497 PMC7119170

[3] Xin Yu J Hubbard-Lucey VM Tang J. Immuno-oncology drug development goes global. Nat Rev Drug discovery. (2019) 18(12):899–900. doi: 10.1038/d41573-019-00167-9 31780841

[4] Robert C. A decade of immune-checkpoint inhibitors in cancer therapy. Nat Commun (2020) 11(1):3801. doi: 10.1038/s41467-020-17670-y 32732879 PMC7393098

[5] Locksley RM Killeen N Lenardo MJ. The TNF and TNF receptor superfamilies: integrating mammalian biology. Cell (2001) 104(4):487–501. doi: 10.1016/S0092-8674(01)00237-9 11239407

[6] Song Y Buchwald P. TNF superfamily protein-protein interactions: feasibility of small- molecule modulation. Curr Drug targets. (2015) 16(4):393–408. doi: 10.2174/1389450116666150223115628 25706111 PMC4408546

[7] Ward-Kavanagh LK Lin WW Šedý JR Ware CF. The TNF receptor superfamily in Co-stimulating and Co-inhibitory responses. Immunity (2016) 44(5):1005–19. doi: 10.1016/j.immuni.2016.04.019 PMC4882112 27192566

[8] Vonderheide RH. CD40 agonist antibodies in cancer immunotherapy. Annu Rev Med (2020) 71:47–58. doi: 10.1146/annurev-med-062518-045435 31412220

[9] Rakhmilevich AL Buhtoiarov IN Malkovsky M Sondel PM. CD40 ligation *in vivo* can induce T cell independent antitumor effects even against immunogenic tumors. Cancer immunol immunother: CII. (2008) 57(8):1151–60. doi: 10.1007/s00262-007-0447-4 PMC11031017 18214476

[10] Lee BO Moyron-Quiroz J Rangel-Moreno J Kusser KL Hartson L Sprague F. CD40, but not CD154, expression on b cells is necessary for optimal primary b cell responses. J Immunol (Baltimore Md: 1950). (2003) 171(11):5707–17. doi: 10.4049/jimmunol.171.11.5707 14634078

[11] Björck P Axelsson B Paulie S. Expression of CD40 and CD43 during activation of human b lymphocytes. Scandinavian J Immunol (1991) 33(2):211–8. doi: 10.1111/j.1365-3083.1991.tb03751.x 1708162

[12] Hernandez MG Shen L Rock KL. CD40 on APCs is needed for optimal programming, maintenance, and recall of CD8+ T cell memory even in the absence of CD4+ T cell help. J Immunol (Baltimore Md: 1950). (2008) 180(7):4382–90. doi: 10.4049/jimmunol.180.7.4382 18354158

[13] Young LS Dawson CW Brown KW Rickinson AB. Identification of a human epithelial cell surface protein sharing an epitope with the C3d/Epstein-Barr virus receptor molecule of b lymphocytes. Int J cancer. (1989) 43(5):786–94. doi: 10.1002/ijc.2910430508 2469656

[14] Altenburg A Baldus SE Smola H Pfister H Hess S. CD40 ligand-CD40 interaction induces chemokines in cervical carcinoma cells in synergism with IFN-gamma. J Immunol (Baltimore Md: 1950). (1999) 162(7):4140–7. doi: 10.4049/jimmunol.162.7.4140 10201939

[15] Cooke PW James ND Ganesan R Wallace M Burton A Young LS. CD40 expression in bladder cancer. J pathol (1999) 188(1):38–43. doi: 10.1002/(SICI)1096-9896(199905)188:1<38::AID-PATH315>3.0.CO;2-B 10398138

[16] Gallagher NJ Eliopoulos AG Agathangelo A Oates J Crocker J Young LS. CD40 activation in epithelial ovarian carcinoma cells modulates growth, apoptosis, and cytokine secretion. Mol pathol: MP. (2002) 55(2):110–20. doi: 10.1136/mp.55.2.110 PMC1187159 11950960

[17] Tan J Town T Mori T Obregon D Wu Y DelleDonne A. CD40 is expressed and functional on neuronal cells. EMBO J (2002) 21(4):643–52. doi: 10.1093/emboj/21.4.643 PMC125862 11847112

[18] Wagner AH Güldenzoph B Lienenlüke B Hecker M. CD154/CD40-mediated expression of CD154 in endothelial cells: consequences for endothelial cell-monocyte interaction. Arteriosclerosis thrombosis Vasc Biol (2004) 24(4):715–20. doi: 10.1161/01.ATV.0000122853.99978.b1 14976003

[19] Cella M Scheidegger D Palmer-Lehmann K Lane P Lanzavecchia A Alber G. Ligation of CD40 on dendritic cells triggers production of high levels of interleukin-12 and enhances T cell stimulatory capacity: T-T help *via* APC activation. J Exp Med (1996) 184(2):747–52. doi: 10.1084/jem.184.2.747 PMC2192696 8760829

[20] Morrison AH Diamond MS Hay CA Byrne KT Vonderheide RH. Sufficiency of CD40 activation and immune checkpoint blockade for T cell priming and tumor immunity. Proc Natl Acad Sci United States America. (2020) 117(14):8022–31. doi: 10.1073/pnas.1918971117 PMC7149500 32213589

[21] Richards DM Sefrin JP Gieffers C Hill O Merz C. Concepts for agonistic targeting of CD40 in immuno-oncology. Hum Vaccines immunotherapeutics. (2020) 16(2):377–87. doi: 10.1080/21645515.2019.1653744 PMC7062441 31403344

[22] Choi Y Shi Y Haymaker CL Naing A Ciliberto G Hajjar J. T-Cell agonists in cancer immunotherapy. J immunother Cancer (2020) 8(2):1–14. doi: 10.1136/jitc-2020-000966 PMC7537335 33020242

[23] Merz C Sykora J Marschall V Richards DM Heinonen K Redondo Müller M. The hexavalent CD40 agonist HERA-CD40L induces T-cell-mediated antitumor immune response through activation of antigen-presenting cells. J immunother (Hagerstown Md: 1997). (2018) 41(9):385–98. doi: 10.1097/CJI.0000000000000246 30273198

[24] Gieffers C Kluge M Merz C Sykora J Thiemann M Schaal R. APG350 induces superior clustering of TRAIL receptors and shows therapeutic antitumor efficacy independent of cross-linking *via* fcγ receptors. Mol Cancer Ther (2013) 12(12):2735–47. doi: 10.1158/1535-7163.MCT-13-0323 24101228

[25] Richards DM Marschall V Billian-Frey K Heinonen K Merz C Redondo Müller M. HERA-GITRL activates T cells and promotes anti-tumor efficacy independent of FcγR-binding functionality. J immunother cancer. (2019) 7(1):191. doi: 10.1186/s40425-019-0671-4 31324216 PMC6642547

[26] Thiemann M Richards DM Heinonen K Kluge M Marschall V Merz C. A single-Chain-Based hexavalent CD27 agonist enhances T cell activation and induces anti-tumor immunity. Front Oncol (2018) 8:387. doi: 10.3389/fonc.2018.00387 30298117 PMC6160747

[27] Legler K Hauser C Egberts JH Willms A Heneweer C Boretius S. The novel TRAIL-receptor agonist APG350 exerts superior therapeutic activity in pancreatic cancer cells. Cell Death disease. (2018) 9(5):445. doi: 10.1038/s41419-018-0478-0 29670075 PMC5906476

[28] Dovedi SJ Adlard AL Ota Y Murata M Sugaru E Koga-Yamakawa E. Intravenous administration of the selective toll-like receptor 7 agonist DSR-29133 leads to anti-tumor efficacy in murine solid tumor models which can be potentiated by combination with fractionated radiotherapy. Oncotarget (2016) 7(13):17035–46. doi: 10.18632/oncotarget.7928 PMC4941369 26959743

[29] Byrne KT Betts CB Mick R Sivagnanam S Bajor DL Laheru DA. Neoadjuvant selicrelumab, an agonist CD40 antibody, induces changes in the tumor microenvironment in patients with resectable pancreatic cancer. Clin Cancer Res (2021) 27(16):4574–86. doi: 10.1158/1078-0432.CCR-21-1047 PMC8667686 34112709

[30] Richman LP Vonderheide RH. Anti-human CD40 monoclonal antibody therapy is potent without FcR crosslinking. Oncoimmunology (2014) 3:e28610. doi: 10.4161/onci.28610 25097801 PMC4091558

[31] Pound JD Challa A Holder MJ Armitage RJ Dower SK Fanslow WC. Minimal cross-linking and epitope requirements for CD40-dependent suppression of apoptosis contrast with those for promotion of the cell cycle and homotypic adhesions in human b cells. Int Immunol (1999) 11(1):11–20. doi: 10.1093/intimm/11.1.11 10050669

[32] Ni CZ Welsh K Leo E Chiou CK Wu H Reed JC. Molecular basis for CD40 signaling mediated by TRAF3. Proc Natl Acad Sci United States America. (2000) 97(19):10395–9. doi: 10.1073/pnas.97.19.10395 PMC27035 10984535

[33] Fang DF He K Wang N Sang ZH Qiu X Xu G. NEDD4 ubiquitinates TRAF3 to promote CD40-mediated AKT activation. Nat Commun (2014) 5:4513. doi: 10.1038/ncomms5513 25072696

[34] Hostager BS Kashiwada M Colgan JD Rothman PB. HOIL-1L interacting protein (HOIP) is essential for CD40 signaling. PloS One (2011) 6(8):e23061. doi: 10.1371/journal.pone.0023061 21829693 PMC3148254

[35] Elgueta R Benson MJ de Vries VC Wasiuk A Guo Y Noelle RJ. Molecular mechanism and function of CD40/CD40L engagement in the immune system. Immunol Rev (2009) 229(1):152–72. doi: 10.1111/j.1600-065X.2009.00782.x PMC3826168 19426221

[36] Zadoroznyj A Dubrez L. Cytoplasmic and nuclear functions of cIAP1. Biomolecules (2022) 12(2):1–19. doi: 10.3390/biom12020322 PMC8869227 35204822

[37] Uren AG Pakusch M Hawkins CJ Puls KL Vaux DL. Cloning and expression of apoptosis inhibitory protein homologs that function to inhibit apoptosis and/or bind tumor necrosis factor receptor-associated factors. Proc Natl Acad Sci United States America. (1996) 93(10):4974–8. doi: 10.1073/pnas.93.10.4974 PMC39390 8643514

[38] Dueber EC Schoeffler AJ Lingel A Elliott JM Fedorova AV Giannetti AM. Antagonists induce a conformational change in cIAP1 that promotes autoubiquitination. Sci (New York NY). (2011) 334(6054):376–80. doi: 10.1126/science.1207862 22021857

[39] Bishop GA Stunz LL Hostager BS. TRAF3 as a multifaceted regulator of b lymphocyte survival and activation. Front Immunol (2018) 9:2161. doi: 10.3389/fimmu.2018.02161 30319624 PMC6165887

[40] Pomerantz JL Baltimore D. NF-kappaB activation by a signaling complex containing TRAF2, TANK and TBK1, a novel IKK-related kinase. EMBO J (1999) 18(23):6694–704. doi: 10.1093/emboj/18.23.6694 PMC1171732 10581243

[41] Azeem W Bakke RM Appel S Øyan AM Kalland KH. Dual pro- and anti-inflammatory features of monocyte-derived dendritic cells. Front Immunol (2020) 11:438. doi: 10.3389/fimmu.2020.00438 32292402 PMC7120039

[42] Schulz D Severin Y Zanotelli VRT Bodenmiller B. In-depth characterization of monocyte-derived macrophages using a mass cytometry-based phagocytosis assay. Sci Rep (2019) 9(1):1925. doi: 10.1038/s41598-018-38127-9 30760760 PMC6374473

[43] Hirano A Longo DL Taub DD Ferris DK Young LS Eliopoulos AG. Inhibition of human breast carcinoma growth by a soluble recombinant human CD40 ligand. Blood (1999) 93(9):2999–3007. doi: 10.1182/blood.V93.9.2999 10216096

[44] Bossen C Ingold K Tardivel A Bodmer JL Gaide O Hertig S. Interactions of tumor necrosis factor (TNF) and TNF receptor family members in the mouse and human. J Biol Chem (2006) 281(20):13964–71. doi: 10.1074/jbc.M601553200 16547002

[45] Sato Y Fu Y Liu H Lee MY Shaw MH. Tumor-immune profiling of CT-26 and colon 26 syngeneic mouse models reveals mechanism of anti-PD-1 response. BMC cancer. (2021) 21(1):1222. doi: 10.1186/s12885-021-08974-3 34774008 PMC8590766

[46] Philippou Y Sjoberg HT Murphy E Alyacoubi S Jones KI Gordon-Weeks AN. Impacts of combining anti-PD-L1 immunotherapy and radiotherapy on the tumour immune microenvironment in a murine prostate cancer model. Br J cancer. (2020) 123(7):1089–100. doi: 10.1038/s41416-020-0956-x PMC7525450 32641865

[47] Walshaw RC Honeychurch J Illidge TM Choudhury A. The anti-PD-1 era - an opportunity to enhance radiotherapy for patients with bladder cancer. Nat Rev Urology. (2018) 15(4):251–9. doi: 10.1038/nrurol.2017.172 29089607

[48] Dovedi SJ Lipowska-Bhalla G Beers SA Cheadle EJ Mu L Glennie MJ. Antitumor efficacy of radiation plus immunotherapy depends upon dendritic cell activation of effector CD8+ T cells. Cancer Immunol Res (2016) 4(7):621–30. doi: 10.1158/2326-6066.CIR-15-0253 PMC5348028 27241845

[49] Sun SC. The noncanonical NF-κB pathway. Immunol Rev (2012) 246(1):125–40. doi: 10.1111/j.1600-065X.2011.01088.x PMC3313452 22435551

[50] Liao G Zhang M Harhaj EW Sun SC. Regulation of the NF-kappaB-inducing kinase by tumor necrosis factor receptor-associated factor 3-induced degradation. J Biol Chem (2004) 279(25):26243–50. doi: 10.1074/jbc.M403286200 15084608

[51] Fotin-Mleczek M Henkler F Hausser A Glauner H Samel D Graness A. Tumor necrosis factor receptor-associated factor (TRAF) 1 regulates CD40-induced TRAF2-mediated NF-kappaB activation. J Biol Chem (2004) 279(1):677–85. doi: 10.1074/jbc.M310969200 14557256

[52] Tsitsikov EN Laouini D Dunn IF Sannikova TY Davidson L Alt FW. TRAF1 is a negative regulator of TNF signaling. enhanced TNF signaling in TRAF1-deficient mice. Immunity (2001) 15(4):647–57. doi: 10.1016/S1074-7613(01)00207-2 11672546

[53] Arron JR Pewzner-Jung Y Walsh MC Kobayashi T Choi Y. Regulation of the subcellular localization of tumor necrosis factor receptor-associated factor (TRAF)2 by TRAF1 reveals mechanisms of TRAF2 signaling. J Exp Med (2002) 196(7):923–34. doi: 10.1084/jem.20020774 PMC2194023 12370254

[54] Abdul-Sater AA Edilova MI Clouthier DL Mbanwi A Kremmer E Watts TH. The signaling adaptor TRAF1 negatively regulates toll-like receptor signaling and this underlies its role in rheumatic disease. Nat Immunol (2017) 18(1):26–35. doi: 10.1038/ni.3618 27893701

[55] Bishop GA Abdul-Sater AA Watts TH. Editorial: TRAF proteins in health and disease. Front Immunol (2019) 10:26. doi: 10.3389/fimmu.2019.00326 30863413 PMC6400096

[56] Ibraheem K Yhmed AMA Nasef MM Georgopoulos NT. TRAF3/p38-JNK signalling crosstalk with intracellular-TRAIL/Caspase-10-Induced apoptosis accelerates ROS-driven cancer cell-specific death by CD40. Cells (2022) 11(20):1–19. doi: 10.3390/cells11203274 PMC9600997 36291141

[57] Workman LM Zhang L Fan Y Zhang W Habelhah H. TRAF2 ser-11 phosphorylation promotes cytosolic translocation of the CD40 complex to regulate downstream signaling pathways. Mol Cell Biol (2020) 40(9):1–19. doi: 10.1128/MCB.00429-19 PMC7156217 32041822

[58] Shi JH Sun SC. Tumor necrosis factor receptor-associated factor regulation of nuclear factor κB and mitogen-activated protein kinase pathways. Front Immunol (2018) 9:1849. doi: 10.3389/fimmu.2018.01849 30140268 PMC6094638

[59] Davies CC Mak TW Young LS Eliopoulos AG. TRAF6 is required for TRAF2-dependent CD40 signal transduction in nonhemopoietic cells. Mol Cell Biol (2005) 25(22):9806–19. doi: 10.1128/MCB.25.22.9806-9819.2005 PMC1280261 16260598

[60] Rowland SL Tremblay MM Ellison JM Stunz LL Bishop GA Hostager BS. A novel mechanism for TNFR-associated factor 6-dependent CD40 signaling. J Immunol (Baltimore Md: 1950). (2007) 179(7):4645–53. doi: 10.4049/jimmunol.179.7.4645 17878362

[61] Brown KD Hostager BS Bishop GA. Regulation of TRAF2 signaling by self-induced degradation. J Biol Chem (2002) 277(22):19433–8. doi: 10.1074/jbc.M111522200 11909853

[62] Georgopoulos NT Steele LP Thomson MJ Selby PJ Southgate J Trejdosiewicz LK. A novel mechanism of CD40-induced apoptosis of carcinoma cells involving TRAF3 and JNK/AP-1 activation. Cell Death differentiation. (2006) 13(10):1789–801. doi: 10.1038/sj.cdd.4401859 16429118

[63] Ibraheem K Yhmed AMA Qayyum T Bryan NP Georgopoulos NT. CD40 induces renal cell carcinoma-specific differential regulation of TRAF proteins, ASK1 activation and JNK/p38-mediated, ROS-dependent mitochondrial apoptosis. Cell Death discovery. (2019) 5:148. doi: 10.1038/s41420-019-0229-8 31815003 PMC6892818

[64] Hostager BS Bishop GA. Cutting edge: contrasting roles of TNF receptor-associated factor 2 (TRAF2) and TRAF3 in CD40-activated b lymphocyte differentiation. J Immunol (Baltimore Md: 1950). (1999) 162(11):6307–11. doi: 10.4049/jimmunol.162.11.6307 10352240

[65] Chen Y Song Y Du W Gong L Chang H Zou Z. Tumor-associated macrophages: an accomplice in solid tumor progression. J Biomed science. (2019) 26(1):78. doi: 10.1186/s12929-019-0568-z PMC6800990 31629410

[66] Labani-Motlagh A Ashja-Mahdavi M Loskog A. The tumor microenvironment: a milieu hindering and obstructing antitumor immune responses. Front Immunol (2020) 11:940. doi: 10.3389/fimmu.2020.00940 32499786 PMC7243284

[67] van Dalen FJ van Stevendaal M Fennemann FL Verdoes M Ilina O. Molecular repolarisation of tumour-associated macrophages. Molecules (Basel Switzerland). (2018) 24(1):1–25. doi: 10.3390/molecules24010009 PMC6337345 30577495

[68] Camphausen K Tofilon PJ. Combining radiation and molecular targeting in cancer therapy. Cancer Biol Ther (2004) 3(3):247–50. doi: 10.4161/cbt.3.3.544 15107611

[69] Harrington KJ Billingham LJ Brunner TB Burnet NG Chan CS Hoskin P. Guidelines for preclinical and early phase clinical assessment of novel radiosensitisers. Br J cancer. (2011) 105(5):628–39. doi: 10.1038/bjc.2011.240 PMC3188925 21772330

[70] Good JS Harrington KJ. The hallmarks of cancer and the radiation oncologist: updating the 5Rs of radiobiology. Clin Oncol (Royal Coll Radiologists (Great Britain)). (2013) 25(10):569–77. doi: 10.1016/j.clon.2013.06.009 23850153

[71] Laoui D Van Overmeire E De Baetselier P Van Ginderachter JA Raes G. Functional relationship between tumor-associated macrophages and macrophage colony-stimulating factor as contributors to cancer progression. Front Immunol (2014) 5:489. doi: 10.3389/fimmu.2014.00489 25339957 PMC4188035

[72] Barker HE Paget JT Khan AA Harrington KJ. The tumour microenvironment after radiotherapy: mechanisms of resistance and recurrence. Nat Rev Cancer. (2015) 15(7):409–25. doi: 10.1038/nrc3958 PMC4896389 26105538

[73] Beatty GL Chiorean EG Fishman MP Saboury B Teitelbaum UR Sun W. CD40 agonists alter tumor stroma and show efficacy against pancreatic carcinoma in mice and humans. Sci (New York NY). (2011) 331(6024):1612–6. doi: 10.1126/science.1198443 PMC3406187 21436454

[74] Ying L Yan F Xu D. Cancer patient stratification based on the tumor microenvironment. J Thorac disease. (2020) 12(8):4522–6. doi: 10.21037/jtd.2020.03.77 PMC7475598 32944367

[75] Halama N. The next age of immunotherapy: optimisation, stratification and therapeutic synergies. Br J cancer. (2019) 120(1):1–2. doi: 10.1038/s41416-018-0330-4 30413823 PMC6325151

[76] Fang C Xu D Su J Dry JR Linghu B. DeePaN: deep patient graph convolutional network integrating clinico-genomic evidence to stratify lung cancers for immunotherapy. NPJ digital Med (2021) 4(1):14. doi: 10.1038/s41746-021-00381-z PMC7854753 33531613

