# Supplementary figures and images for: The CD40 agonist HERA-CD40L results in enhanced activation of antigen presenting cells, promoting an anti-tumor effect alone and in combination with radiotherapy
Source: Front Immunol. 2023 May 26;14:1160116. doi: 10.3389/fimmu.2023.1160116 (PMC10251205; doi:10.3389/fimmu.2023.1160116)

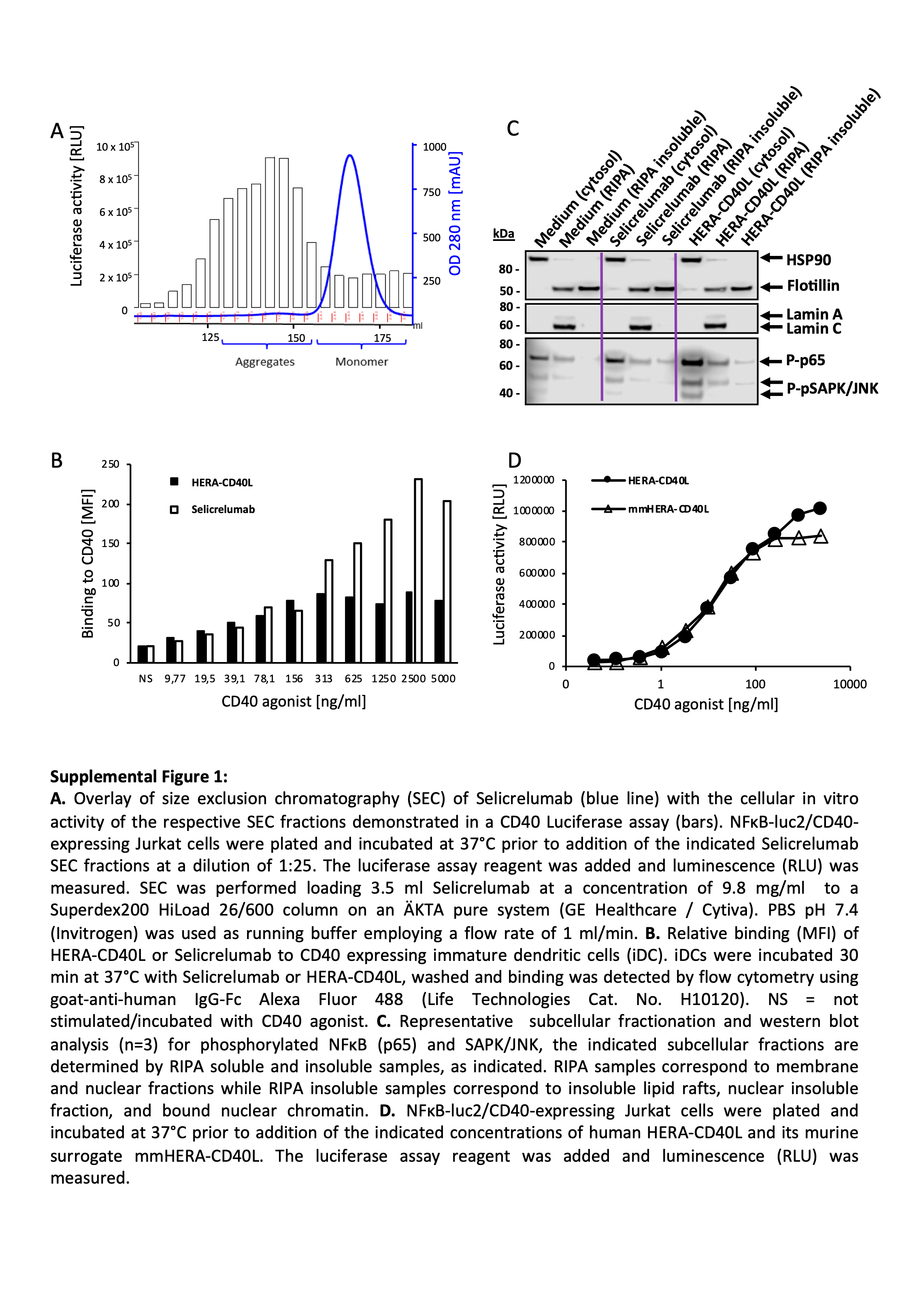

Supplement: Supplementary file 1 [file Image_1.tiff]

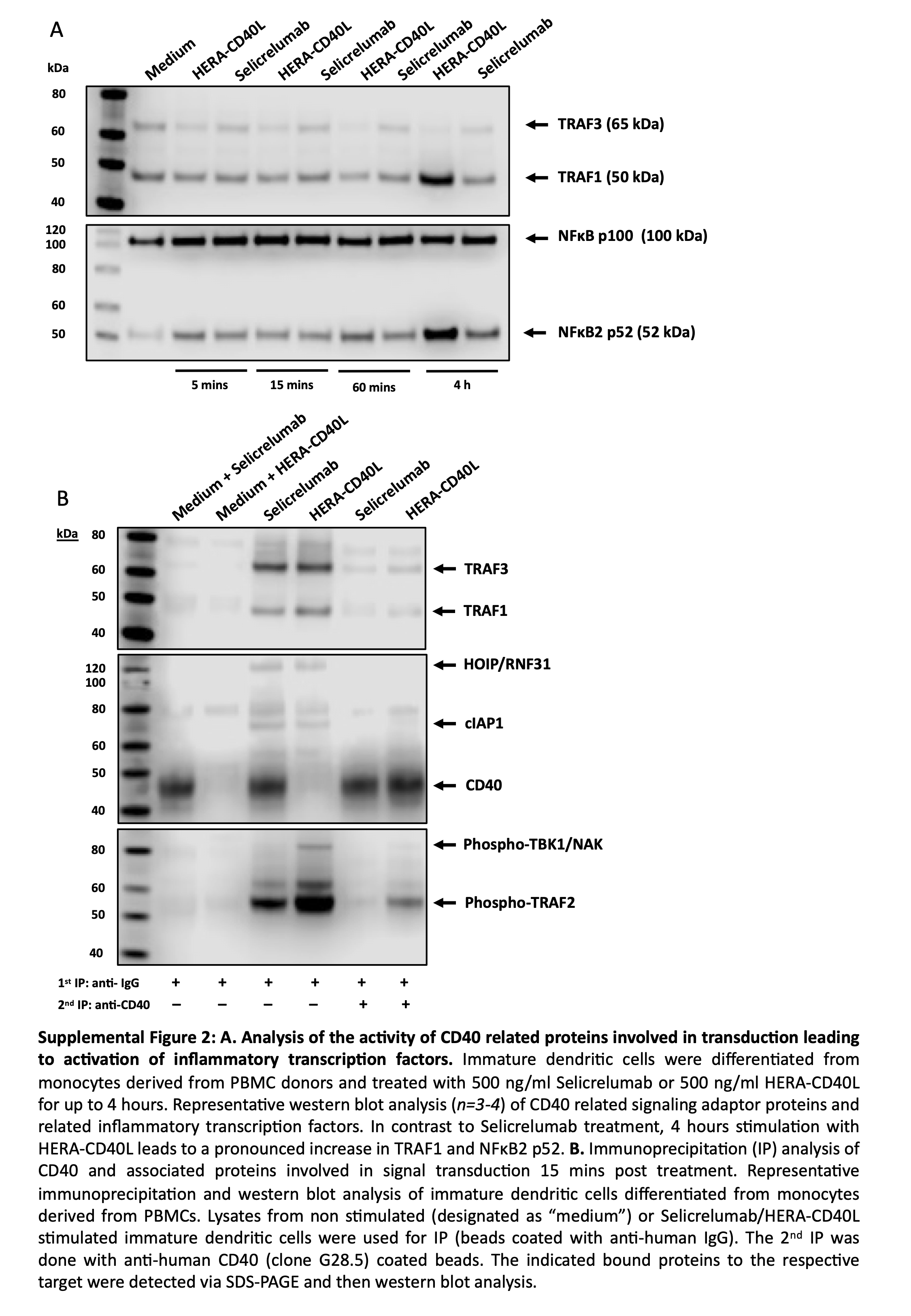

Supplement: Supplementary file 2 [file Image_2.tiff]

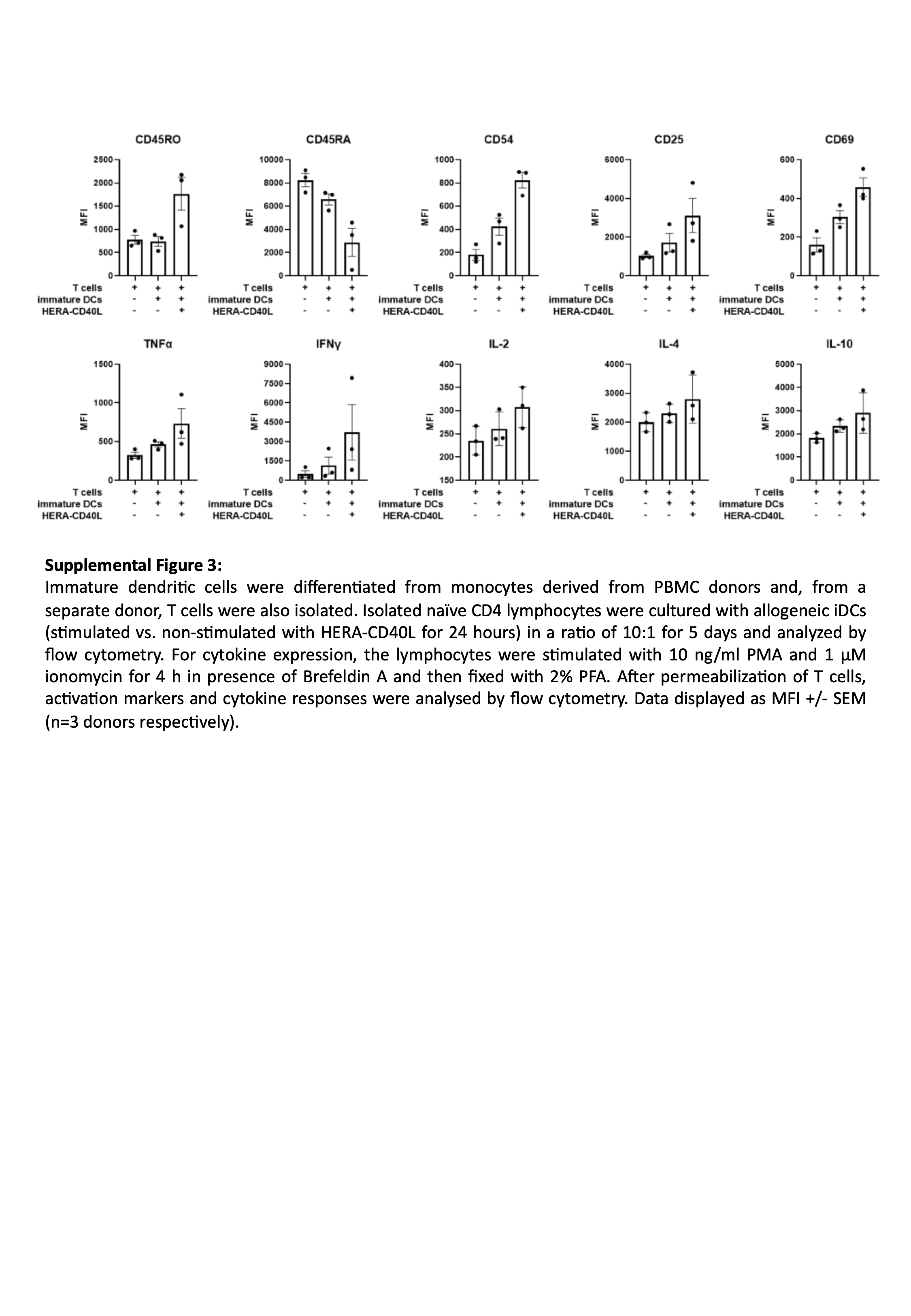

Supplement: Supplementary file 3 [file Image_3.tiff]

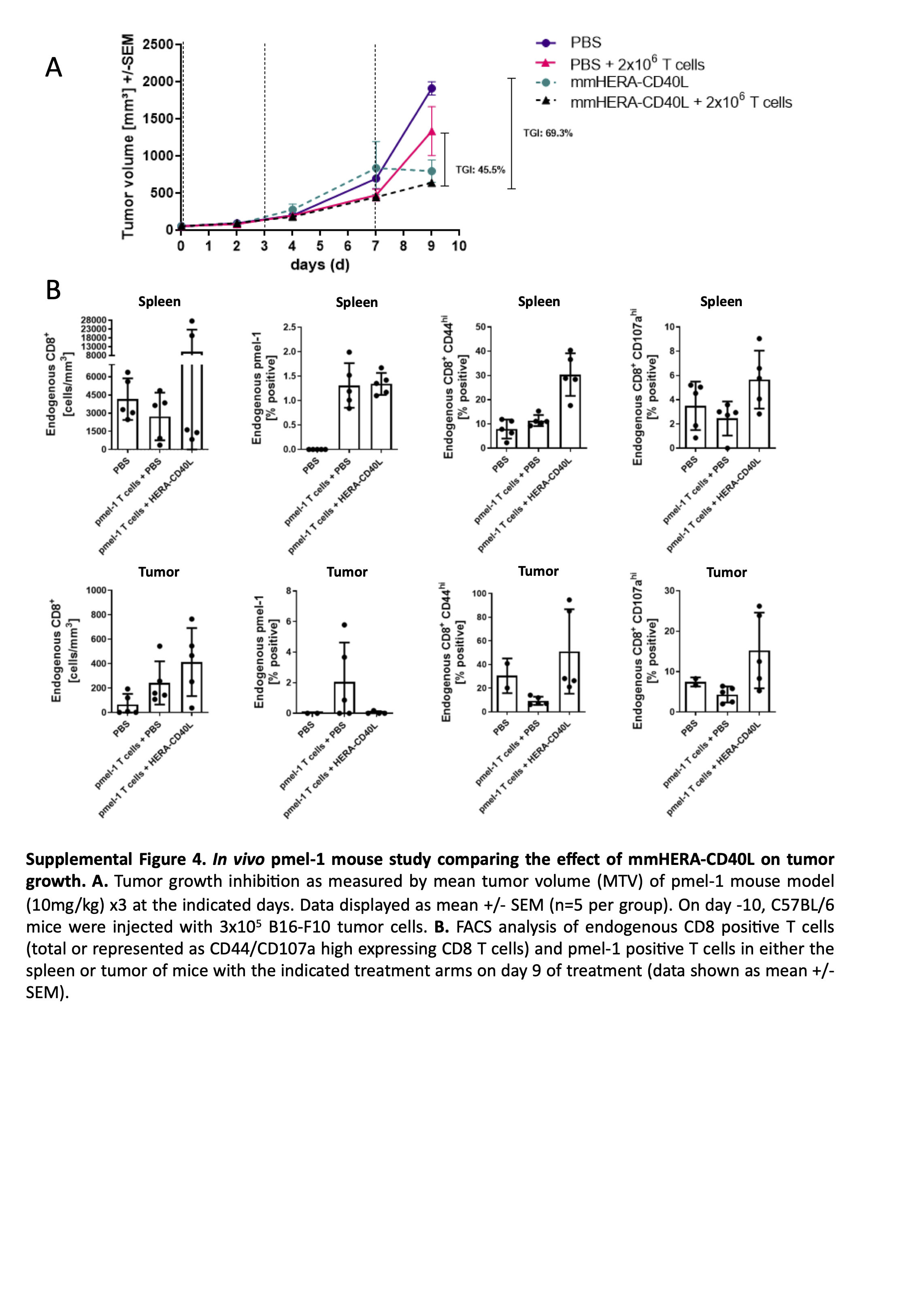

Supplement: Supplementary file 4 [file Image_4.tiff]

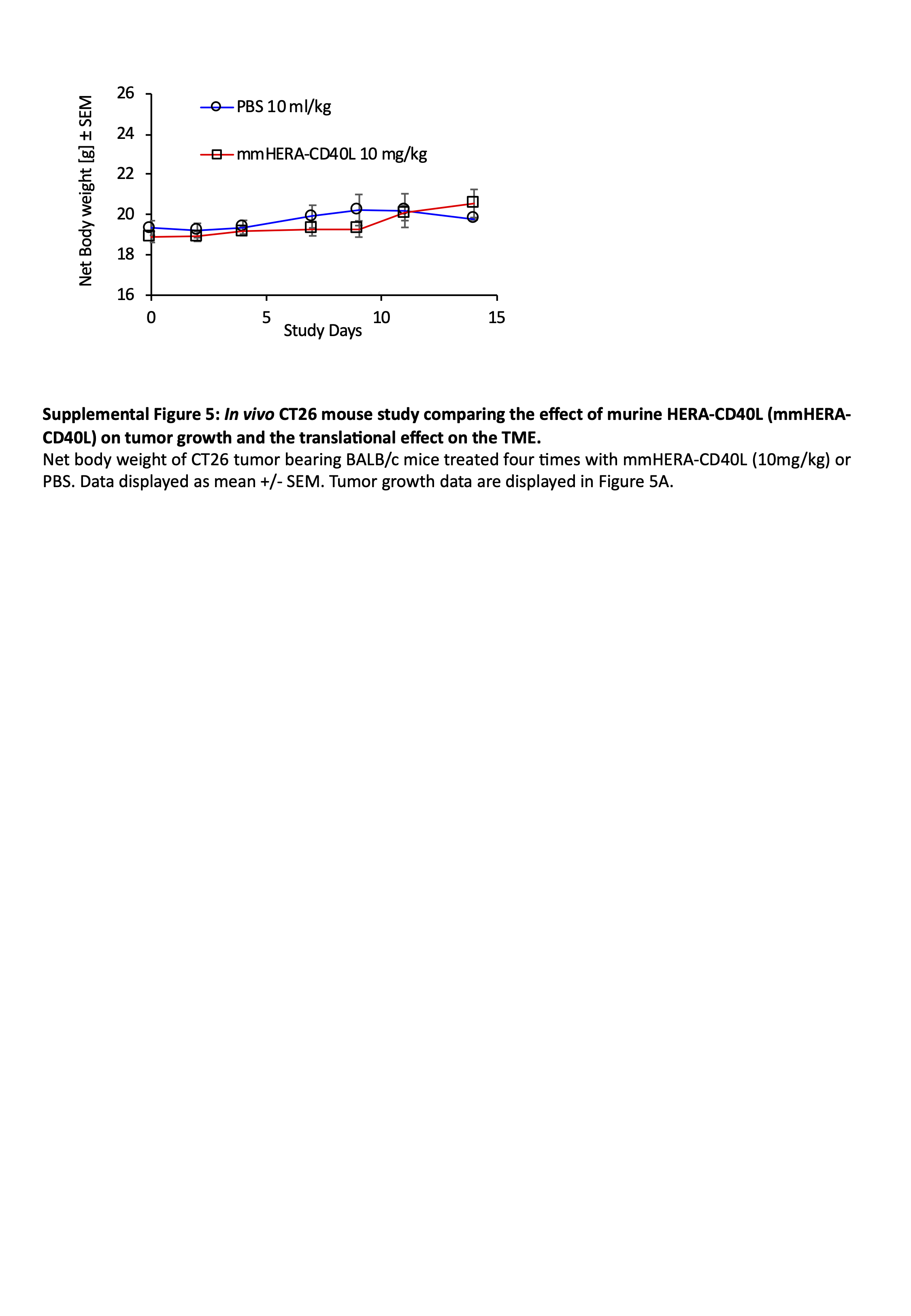

Supplement: Supplementary file 5 [file Image_5.tiff]

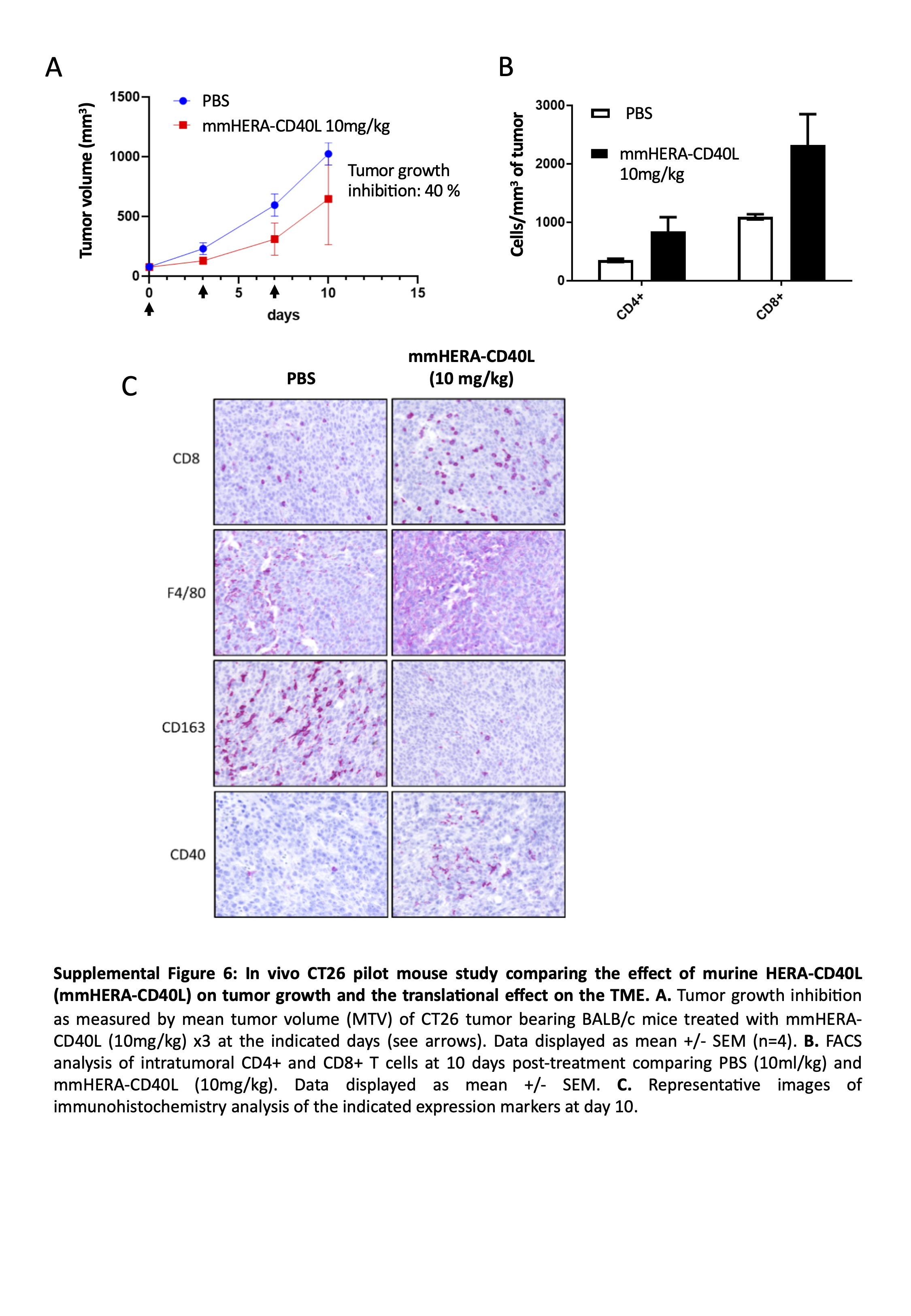

Supplement: Supplementary file 6 [file Image_6.tiff]

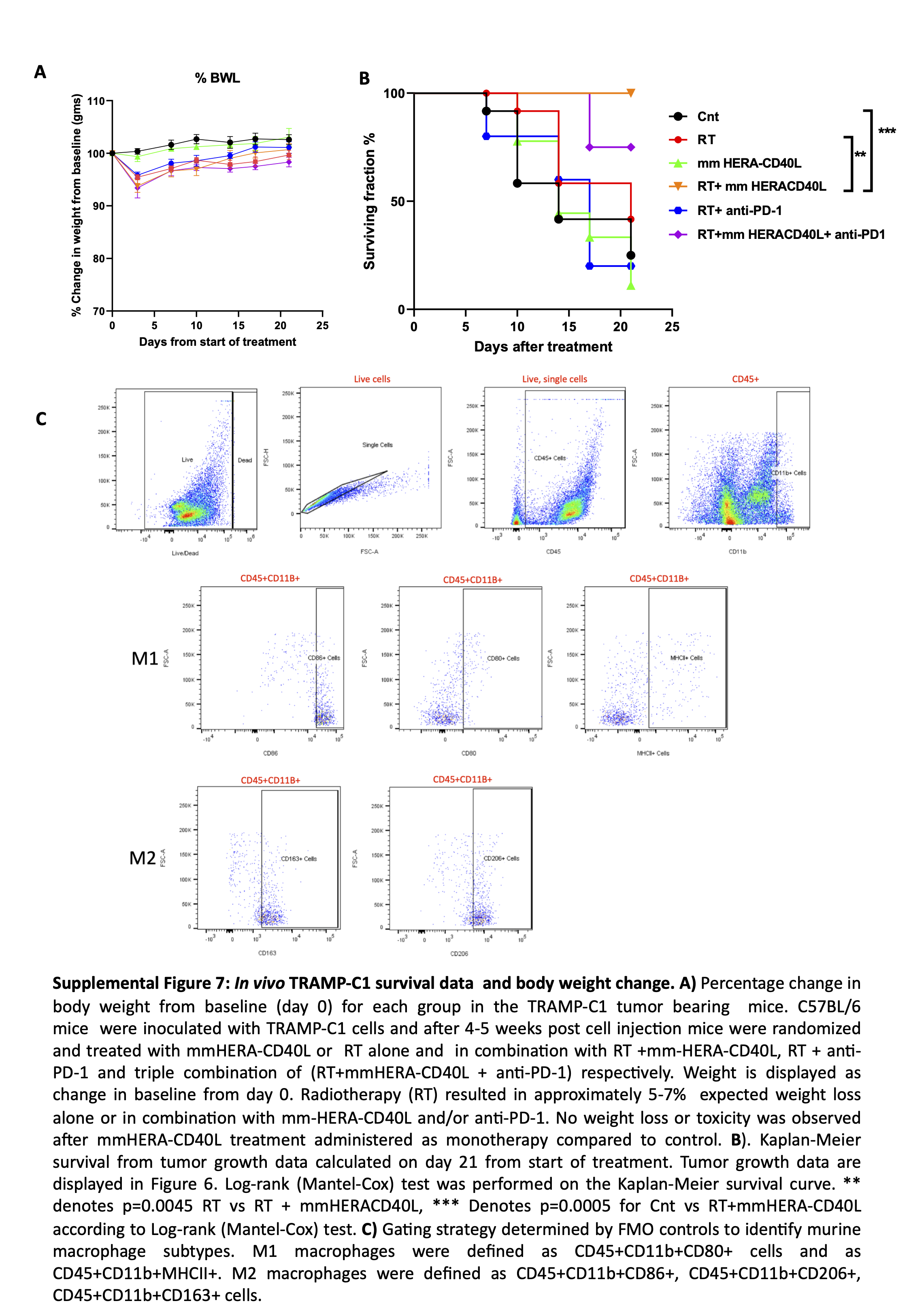

Supplement: Supplementary file 7 [file Image_7.tiff]

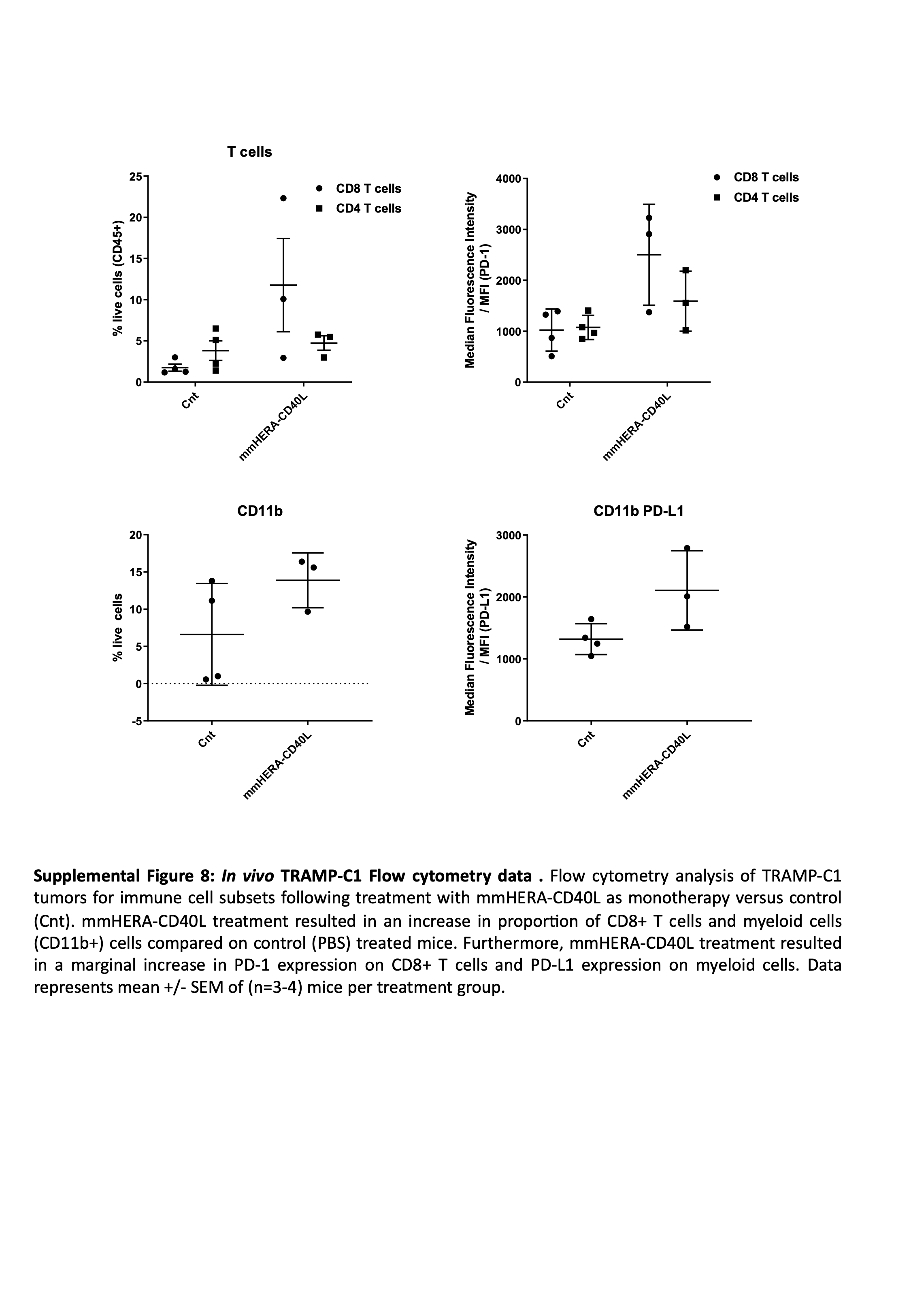

Supplement: Supplementary file 8 [file Image_8.tiff]
