# Supplementary material for: The CD40 agonist HERA-CD40L results in enhanced activation of antigen presenting cells, promoting an anti-tumor effect alone and in combination with radiotherapy
Source: Front Immunol. 2023 May 26;14:1160116. doi: 10.3389/fimmu.2023.1160116 (PMC10251205; doi:10.3389/fimmu.2023.1160116)
Supplement: Supplementary file 9 [file Table_1.docx]

## Tables

### Table 1

| **Antibody** | **Company** | **Cat-No.** | **Target species** | **Origin Species** | **Clone** |
| --- | --- | --- | --- | --- | --- |
| CD11b | Abcam | ab133357 | Human, Mouse | Rabbit | EPR1344 |
| CD11c | Cell Signaling | 97585 | Mouse | Rabbit | D1V9Y |
| CD163 | Abcam | ab182422 | Human, Mouse | Rabbit | EPR19518 |
| CD197 (CCR7) Alex Fluor 488 | BioLegend | 353206 | Human | Mouse | G043H7 |
| CD25 APC | BioLegend | 302610 | Human | Mouse | BC96 |
| CD274 (PD-L1) PE | BioLegend | 329706 | Human | Mouse | 29E.2A3 |
| CD4 | Cell Signaling | 25229 | Mouse | Rabbit | D7D2Z |
| CD4 PE/Cy7 | BioLegend | 344612 | Human | Mouse | SK3 |
| CD40 (D8W3N) | Cell Signaling | 40868 | Human | Rabbit | D8W3N |
| CD40 (E2Z7J) | Cell Signaling | 86165 | Mouse | Rabbit | E2Z7J |
| CD40 APC | BioLegend | 334308 | Human | Mouse | 5C3 |
| CD45RA APC | BioLegend | 304112 | Human | Mouse | HI100 |
| CD45RO PE | BioLegend | 304206 | Human | Mouse | UCHL1 |
| CD54 Alexa Fluor 488 | BioLegend | 322714 | Human | Mouse | HCD54 |
| CD54/ICAM-1 (E3Q9N) XP® | Cell Signaling | 67836T | Human | Rabbit | E3Q9N |
| CD69 PE | BioLegend | 310906 | Human | Mouse | FN50 |
| CD8 APC/Cy7 | BioLegend | 344714 | Human | Mouse | SK1 |
| CD80 Alexa Fluor 488 | BioLegend | 305214 | Human | Mouse | 2D10 |
| CD83 APC | BioLegend | 305312 | Human | Mouse | HB15e |
| CD86 PE | BioLegend | 305406 | Human | Mouse | IT2.2 |
| CD8a | Cell Signaling | 98941 | Mouse | Rabbit | D4W2Z |
| CD95 (Fas) APC | BioLegend | 305612 | Human | Mouse | DX2 |
| c-IAP1 | Abcam | ab154525 | Human | Rabbit | poly |
| c-IAP1 (D5G9) | Cell Signaling | 7065 | Human | Rabbit | D5G9 |
| F4/80 | BioRad | MCA497G | Mouse | Rat | Cl:A3-1 |
| F4/80 | BioLegend | 123103 | Mouse | Rat | BM8 |
| Fas (CD95) | Cell Signaling | 4233S | Human | Rabbit | C18C12 |
| Flotillin-2 | Cell Signaling | 3436S | Human, Mouse | Rabbit | C42A3 |
| FoxP3 | Cell Signaling | 12653 | Mouse | Rabbit | D608R |
| Histone H3 | Cell Signaling | 9715S | Human, Mouse | Rabbit | poly |
| HLA-DR APC | BioLegend | 361610 | Human | Mouse | Tü36 |
| HOIP/RNF31 (E6M5B) | Cell Signaling | 99633 | Human | Rabbit | E6M5B |
| HSP90 | Cell Signaling | 4877S | Human, Mouse | Rabbit | C45G5 |
| IFN-gamma PE | Biolegend | 506507 | Human | Mouse | B27 |
| IL-10 PE | Biolegend | 506804 | Human | Rat | JES3-19F1 |
| IL-12 PE | Biolegend | 501807 | Human | Mouse | C11.5 |
| IL-13 APC | Biolegend | 501907 | Human | Rat | JES10-5A2 |
| IL-2 Alexa Fluor 488 | Biolegend | 500314 | Human | Rat | MQ1-17H12 |
| IL-21 PE | eBioscience | 12-7219-42 | Human | Mouse | eBioA3-N2 |
| IL-4 APC | eBioscience | 17-7049-42 | Human | Mouse | 8D4-8 |
| IL-8 Alexa Fluor 488 | Biolegend | 511412 | Human | Mouse | E8N1 |
| Isotype control mouse IgG1 Alexa Fluor 647 | R&D | IC002R | n.a. | Mouse | 11711 |
| Isotype control mouse IgG1 Alexa Fluor488 | R&D | IC002G | n.a. | Mouse | 11711 |
| Isotype control mouse IgG1 APC | R&D | IC002A | n.a. | Mouse | 11711 |
| Isotype control Mouse IgG1 APC/Cy7 | BioLegend | 400128 | n.a. | Mouse | MOPC-21 |
| Isotype control mouse IgG1 PE | R&D | IC002P | n.a. | Mouse | 11711 |
| Isotype control Mouse IgG1 PE/Cy7 | eBioscience | 25-4714-42 | n.a. | Mouse | P3.6.2.8.1 |
| Lamin A/C | Cell Signaling | 2032S | Human, Mouse, Rat | Rabbit | poly |
| Lamin B1 (D4Q4Z) | Cell Signaling | 12586S | Human, Mouse, Rat | Rabbit | D4Q4Z |
| Ly-6C | Bio Rad | MCA2389GA | Mouse | Rat | ER-MP20 |
| Ly-6C | BioLegend | 128001 | Mouse | Rat | HK1.4 |
| Ly-6G | BioLegend | 127601 | Mouse | Rat | 1A8 |
| Ly-6G / Ly-6C (Gr-1) | BioLegend | 108402 | Mouse | Rat | RB6-8C5 |
| MCP-1 Alexa Fluor 647 | BD Biosciences | 563496 | Human | Mouse | 5D3-F7 |
| NF-kB p65 | BD Biosciences | 610868 | Human, Rat, Dog, Frog, Rabbit | Mouse | 20/NF-kB/p65 |
| NF-κB1 p105/p50 | Cell Signaling | 3035 S | Human | Rabbit | poly |
| NF-κB2 p100/p52 | Cell Signaling | 37359S | Human | Rabbit | D7A9K |
| P-NF-κB p65 (pS529) Alexa Fluor 488 | BD Biosciences | 558421 | Human | Mouse | K10-895.12.50 |
| p38 | BD Biosciences | 612169 | Human, Mouse, Rat | Mouse | 27/p38a/SAPK2a |
| p44/42 MAPK (Erk1/2) | ThermoFisher | 44-654G | Human, Mouse, Rat | Rabbit | poly |
| P-AKT (pS473) Alex Fluor 488 | BD Biosciences | 560404 | Human, Mouse | Mouse | M89-61 |
| PD-1 | Cell Signaling | 84651 | Mouse | Rabbit | D7D5W |
| P-ERK 1,2 (pT202/pY204) Alex Fluor 488 | BD Biosciences | 612592 | Human, Mouse, Rat | Mouse | A20 |
| Phospho-p38 MAPK (pT180/pY182) | BD Biosciences | 612288 | Human, Mouse, Rat | Mouse | 36/p38 (pT180/pY182) |
| Phospho-p44/42 MAPK (Erk1/2) (Thr202/Tyr204) | Cell Signaling | 9101 S | Human, Mouse, Rat | Rabbit | poly |
| Phospho-SAPK/JNK (Thr183/Tyr185) (81E11) | Cell Signaling | 4668 S | Human, Mouse, Rat | Rabbit | 81E11 |
| Phospho-TAK1 (Ser412) Antibody | Cell Signaling | 9339 S | Human, Mouse, Rat | Rabbit | poly |
| Phospho-TBK1/NAK (Ser172) (D52C2) XP® | Cell Signaling | 5483 S | Human, Mouse | Rabbit | D52C2 |
| Phospho-TRAF2 (Ser11) (E2B6L) | Cell Signaling | 13908 | Human, Mouse | Rabbit | E2B6L |
| P-JNK (pT183/pY185) PE | BD Biosciences | 562480 | Human, Mouse | Mouse | N9-66 |
| Pospho-NF-kB p65 (Ser536) (93H1) | Cell Signaling | 3033 | Human, Mouse, Rat | Rabbit | 93H1+G4G3:G43 |
| P-p38 (Thr180, Tyr182) APC | Invitrogen | 17-9078-42 | Human, Mouse | Mouse | 4NIT4KK |
| P-pSTAT 1 (Ser727) PE | Biolegend | 686404 | Human, Mouse | Mouse | A15158B |
| SAPK/JNK (JNK1 / JNK2) | BD Biosciences | 554285 | Human | Mouse | G151-666 |
| SP1 | Cell Signaling | 9389S | Human | Rabbit | D4C3 |
| TAB1 (C25E9) | Cell Signaling | 3226S | Human | Rabbit | C25E9 |
| TAB2 (C88H10) | Cell Signaling | 3745S | Human, Mouse, Rat | Rabbit | C88H10 |
| TAK1 (D94D7) | Cell Signaling | 5206 S | Human, Mouse, Rat | Rabbit | D94D7 |
| TBK1/NAK | Cell Signaling | 3013 S | Human, Mouse, Rat | Rabbit | poly |
| TNF-a Alexa Fluor 488 | Biolegend | 502915 | Human | Mouse | MAb11 |
| TRAF1 (45D3) | Cell Signaling | 4715 | Human | Rabbit | 45D3 |
| TRAF2 | Cell Signaling | 4724 | Human, Mouse | Rabbit | poly |
| TRAF2 | ThermoFisher | 711288 | Human, Mouse, Rat | Rabbit | 12HCLC |
| TRAF2 antibody [EPR7064] | Abcam | ab167163 | Human | Rabbit | EPR7064 |
| TRAF3 (D1N5B) | Cell Signaling | 61095 | Human | Rabbit | D1N5B |
| TRAF6 (D21G3) | Cell Signaling | 8028 | Human | Rabbit | D21G3 |
